# Alignment, Anticipation, Adaptation, or Lagging Behind? Age-Based Regulations in Assisted Reproduction and Late Fertility

**DOI:** 10.1111/padr.12658

**Published:** 2024-09-30

**Authors:** Marie-Caroline Compans

**Affiliations:** University of Vienna, Wittgenstein Centre for Demography and Global Human Capital (IIASA, OeAW, University of Vienna)

## Abstract

This paper focuses on age restrictions on access to infertility treatments and eligibility for their public reimbursement, exploring their relevancy in contexts of rising late birth rates (40+). I explore how age-based reimbursement policies for in vitro fertilization treatments have responded to these fertility trends in 27 high-income countries and in which regulatory frameworks for medically assisted reproduction (MAR) very late births (45+) have particularly increased. First, I show that while age limits for treatment reimbursement are well aligned with the prevalence of late fertility in some national contexts, in most countries, strict age restrictions are lagging behind the rise in late births. In others, pronatalist policies have prompted permissive age criteria or law revisions, anticipating or adapting to rising trends in late births. Second, the rise in very late births has been limited in some contexts with strict age-based rules. However, the analysis suggests that the impact of MAR on very late births may also be influenced by contextual factors other than regulations.

## Introduction

In the wake of major advancements in in vitro fertilization (IVF) and related technologies since the 1980s, the utilization of medically assisted reproduction (MAR) has risen in high-income countries (Smeenk et al. 2023). MAR refers to all modern medical interventions (Zegers-Hochschild et al. 2017) which were originally developed to help couples with specific medical fertility impairments (Wang and Sauer 2006). These technological developments coincided with the tendency to postpone births (Nathan and Pardo 2019), particularly evident in the rise of births at the maternal age of 40 or over (Beaujouan 2020). This demographic trend is linked to broader socioeconomic changes, notably education expansion, partnership instability, and economic uncertainty (Mills et al. 2011).

While the development of MAR and trends in later births were at first independent phenomena, driven by distinct factors, they have progressively become intertwined. Individuals who delay parenthood are more likely to encounter difficulties in conceiving, particularly from their thirties, hence prompting a growing number of people to seek medical help (Adamson 2009). Consequently, high-income countries have witnessed a significant rise in the demand for infertility treatments. At the same time, MAR availability may have also facilitated the postponement of births by enhancing fecundity at later ages (Abramowitz 2017). All in all, the number of births conceived through assisted reproduction is growing (Smeenk et al. 2023) and, presently, 4–7 percent of all domestic births are attributed to MAR procedures in contexts like Czechia (Kocourková, Št’astná, and Burcin 2023), France (La Rochebrochard 2022), Australia (Lazzari, Gray, and Chambers 2021), Spain and Denmark (Devolder and Borisova 2022). The MAR impact on late birth rates has also been shown to be significant in countries like Norway (Goisis et al. 2020), Australia (Lazzari, Gray, and Chambers 2021), the United States (Tierney and Guzzo 2023), and Czechia (Kocourková, Št’astná, and Burcin 2023). However, there are cross-national variations in the demographic relevancy of MAR, which greatly depends on the availability of medical interventions. This is primarily shaped by prevailing regulatory frameworks (Devolder and Borisova 2022; Präg and Mills 2017b).

Establishing legal frameworks to govern medical practice has been a concern since the inception of MAR, albeit it progressed at different paces across countries (Griessler et al. 2022). Assisted reproduction regulations typically address three key areas: permitted techniques, who is granted access, and eligibility criteria for public reimbursement (Engeli and Rothmayr Allison 2016). Techniques have different potentials in addressing the decline of fecundity with age. Typically, success rates for homologous infertility treatments (i.e., without gamete donation) decline with age (Alviggi et al. 2009; Kocourková, Burcin, and Kucera 2014), while oocyte donation (OD) and planned oocyte cryopreservation (POC) show more potential for addressing female age-related infertility when eggs are retrieved at young ages (Ethics Committee of the American Society for Reproductive Medicine 2016; Vos and Blockeel 2018). In addition, regulations often encompass age limits to access treatments (direct age rules) and to be eligible for reimbursement (indirect age rules) (Calhaz-Jorge et al., 2020; Engeli and Rothmayr Allison, 2016).

Across Europe and high-income countries in general, the landscape of these age-based regulations is very diverse (Berg Brigham, Cadier, and Chevreul 2013; Calhaz-Jorge et al. 2020; Präg and Mills 2017a). The rationales behind specific MAR policies are multifaceted and country specific. Although there have always been debates revolving around age-based restrictions, their discussion may have become increasingly relevant as late birth prevalence has risen and as the increasing use of egg donation can potentially offset the decline of IVF success rates with age (Zweifel et al. 2020). By limiting or favoring births at advanced reproductive ages, age-based regulations are also likely to influence late fertility levels (Abramowitz 2017; Bergsvik, Fauske, and Hart 2021). However, so far, little is known about the relationship between age-based rules for MAR access and fertility trends. Are countries with permissive age-based MAR regulations contexts where births have been particularly delayed? Have countries responded to post-poning trends by adapting their legislation? Do restrictive regulations limit the increase in births at advanced ages and do permissive regulations favor them?

This paper addresses these questions by first examining how MAR policies in high-income countries have responded to upward trends in late births. It provides a typology of countries based on the timing of adoption of age-based reimbursement regulations, the degree of permissiveness of adopted age cutoffs, and the evolution of fertility rates from the maternal age of 40. This approach distinguishes between contexts where MAR regulations align with, anticipate, adapt to, or lag behind shifts in fertility schedules towards late ages. Second, within this typology, I examine the contribution of births from age 45 to births from age 40, assuming that most of these very late births are unlikely to occur without medical help. That way, the analysis identifies regulatory contexts where assisted reproduction has the potential to facilitate childbearing at late ages. Eventually, additional regulatory or normative country characteristics are examined to elucidate the cross-national differences in very late birth trends within groups of countries with similar age-based reimbursement regulations.

## Background

### Medically assisted reproduction and later births: A closer bond over time

**FIGURE 1:**
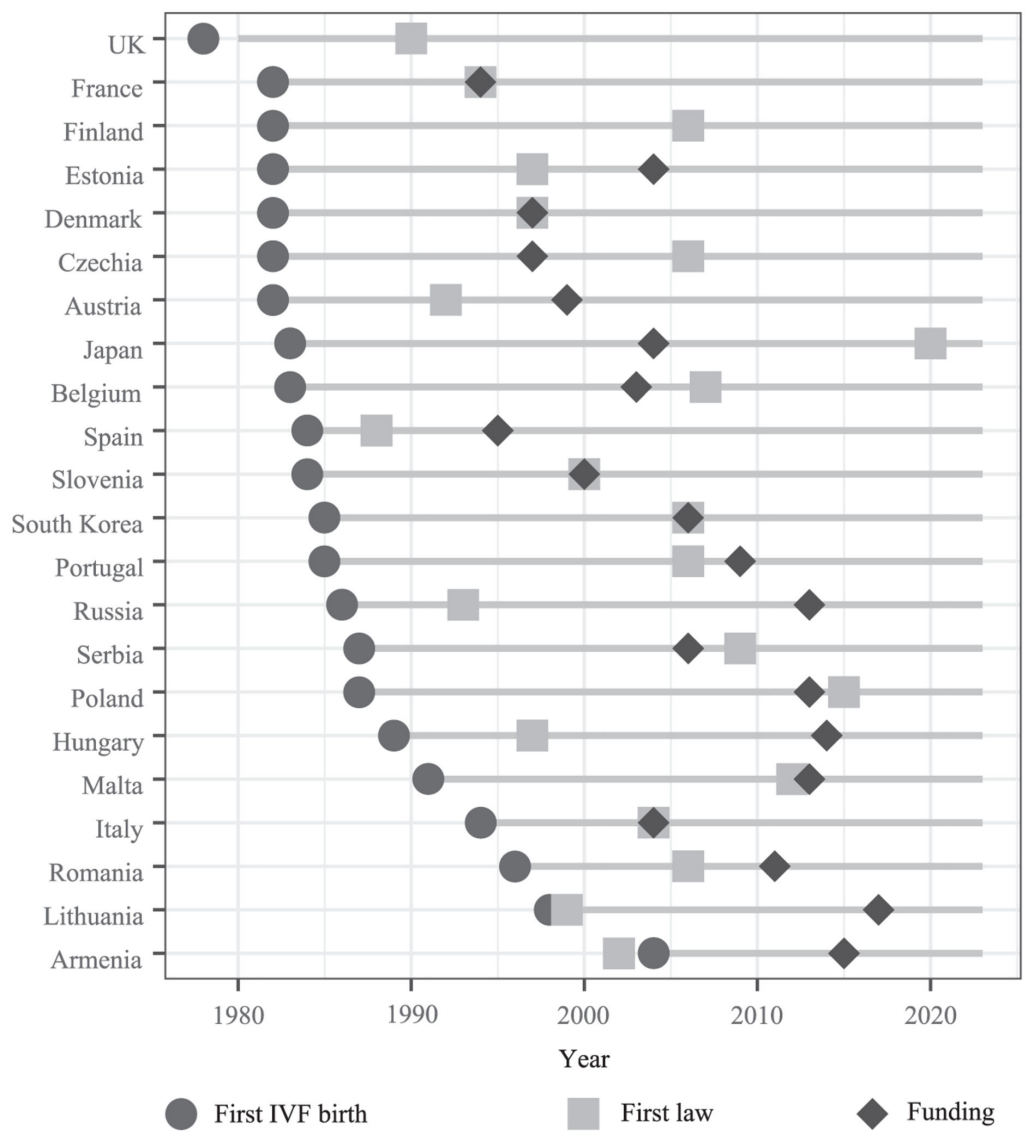
Timeline between the first IVF birth, first MAR legislation, and reimbursement schemes (selected countries) SOURCE: Age-based regulations for assisted reproduction data (collected in 2023).

In the 1980s, high-income countries started to witness the births of the first “test-tube” babies following the birth of Louise Brown in the United Kingdom in 1978 (see Figure 1 in this paper). IVF—which consists of retrieving eggs from a woman’s body and fertilizing them in a laboratory before their implantation in the womb—was initially developed for women with fallopian tube impairments. From this period, OD also emerged as an extension of IVF in which eggs are donated from one woman to another. This medical procedure aimed to prevent premature ovarian failure. Over the following decade, IVF and related techniques benefited from the possibility of cryopreserving gametes. The utilization of this medical intervention rapidly extended to prevent future fertility impairments that would result from medical interventions such as cancer therapy (Wang and Sauer 2006). In essence, MAR technologies were first developed as responses to identified infertility conditions.

Coinciding with these medical advancements, births started to be postponed to later reproductive ages from the late 1970s–1990s onwards depending on the countries (Beaujouan 2020). These fertility trends are explained by economic and social changes, notably educational expansion, female labor force participation, value changes, and transformations in partnership formation and trajectories (Mills et al. 2011). Thus, the factors shaping fertility trends are distinct from those driving MAR development and use.

However, as individuals defer attempts to conceive, they are more likely to find themselves facing difficulties in having a child, prompting some of them to seek medical help. Consequently, postponement trends have resulted in a growing demand for infertility treatment. This delay in childbirth is also evident in the older age profile of female patients, which has progressively extended above the mid-thirties in high-income countries (Adamson 2009; Ben Messaoud, Bouyer, and La Rochebrochard 2020). As reproductive technologies are increasingly used as responses to age-related infertility, MAR usage and later births have become intertwined phenomena. Yet, the extent to which births are postponed in a country is not significantly correlated with the intensity of MAR activity (Devolder and Borisova 2022; Präg and Mills 2017b). Instead, regulatory frameworks are more crucial determinants of cross-national variations in treatment usage, notably because of restrictions to seek medical help to conceive or to be eligible for public reimbursement.

### Regulations of medically assisted reproduction

Regulating the utilization of reproductive technologies can take different forms. MAR can be regulated through guidelines for practitioners (Calhaz-Jorge et al. 2020), which are less obliging than laws. In cases where legislation is scarce, MAR availability may then be mainly shaped by medical practice. Through legislation, medical practice is harmonized at the national level (Martani et al. 2022). Consequently, policies directly influence the availability of infertility treatments. They notably do so by determining which techniques are allowed, who can access them, and who can benefit from public funding of medical interventions (Engeli and Rothmayr Allison 2016).

The regulation of MAR took place at different times and stages of medical practice development across high-income countries. Some have promptly enacted laws shortly after the first IVF babies were conceived (like the United Kingdom, Austria, or France), while others regulated MAR much later (like Italy or Japan) or still have little regulation (like Ireland) (Griessler et al. 2022). As detailed above, MAR technologies were first developed as responses to medical fertility impairments. Therefore, some early regulations of which techniques are allowed—for instance, regarding the legislation of OD—may have been adopted without concerns regarding the demographic impact of assisted reproduction and its potential role in facilitating births at late ages.^1^ In addition, MAR regulations are commonly based on criteria related to the partnership status, sexual orientation, and women’s age (Berg Brigham, Cadier, and Chevreul 2013; Calhaz-Jorge et al. 2020; Engeli and Rothmayr Allison 2016; Präg and Mills 2017a), but given the evolving nature of technologies and their societal implications, laws are subject to revisions. Notably, regulations regarding same-sex couples and single women have shifted towards more permissiveness in Europe (Präg and Mills 2017a), aligning with the evolving acceptance of “new” family forms. However, there has been no thorough examination of the evolution of age-based regulations of assisted reproduction, particularly within a cross-comparative framework.

### Reasons for direct and indirect age-based MAR regulations and their possible revision

There is a constellation of age-based MAR regulations across high-income countries, most of them including no direct age cutoffs to access treatments but a threshold to benefit from their reimbursement (Berg Brigham, Cadier, and Chevreul 2013; Calhaz-Jorge et al. 2020). Indirect age-based rules are particularly relevant to the study of MAR uptake and outcomes as they directly impact the cost of medical procedures for individuals and, in turn, the extent of their usage at a country level (Abramowitz 2017; Bergsvik, Fauske, and Hart 2021; Chambers et al. 2014).

This great diversity in existing age-based regulations for assisted reproduction across high-income contexts is rooted in country-specific rationales that shape either strict or permissive regulatory frameworks. Decision-making processes in this regard often lack transparency (Piek, Pennings, and Provoost 2023), but political scientists have shown that MAR policies are significantly influenced by the interplay between the political system, the level of consensus within the medical community, and participants from the civil society in the political debates—such as women’s or pro-life movements (Engeli and Rothmayr Allison 2016). Policies may thus reflect the historical and cultural contexts in which regulations are debated and adopted, and which arguments have mattered the most then depends on the stake-holders involved in the political decision.

Notably, reasons in favor of restrictive age limits to access treatment reimbursement often stem from a medical perspective, as age greatly organizes gynecological and obstetrical decisions for practitioners (Martani et al. 2022). Motivations are often grounded in the diminishing success rates of MAR and the increased health risks for pregnant women and the fetus with advancing maternal age, leading to greater costs for social security systems (Büchler and Parizer 2017; Mintziori et al. 2013; Vaidya et al. 2015). However, some voices within the medical community have also long supported permissive regulations, relying on the improvements in pregnancy risk screening and advancement in the success rates of reproductive technologies (Antinori et al. 1993; Paulson et al. 2002). Indeed, treatments’ efficacy in compensating for the decline of reproductive capabilities with age is limited (Leridon 2004, 2017), particularly without gamete donation (Alviggi et al. 2009; Beaujouan and Sobotka 2017). However, success rates are higher in heterologous treatments. For instance, according to the Centers for Disease Control and Prevention (CDC) report on Assisted Reproductive Technologies in 2020, live birth rates in the United States declined from 48 percent, 40 percent, 30 percent, and 13 percent from, respectively, ages 30, 35, 40, and 45 for women who used their own eggs. With egg donation, this decrease is smaller from age 30–40 (from 48 percent to 43 percent) and remains around 41 percent from age 40 (Centers for Disease Control and Prevention 2023). Thus, OD (from young donors) and POC (when gametes are preserved at young ages, typically before one’s thirties) hold promise for late births (Ethics Committee of the American Society for Reproductive Medicine 2016; Stoop, Cobo, and Silber 2014; Wang, Farquhar, and Sullivan 2012).

Among other reasons for having specific age restrictions, some proponents also argue for clearly delineated rules for medical practitioners while others are in favor of more autonomy for the medical community (Klitzman 2016; Martani et al. 2022).

On a different note, concerns about the parenting abilities of older parents have also been raised, particularly for mothers who are commonly perceived as primary caregivers (Piek, Pennings, and Provoost 2023). Such an argument may motivate direct or indirect age-based restrictions. Conversely, arguments against age limits assert that older parents have advantaged economic and social resources to rear a child (Barclay and Myrskylä 2016).

MAR regulations are also imbued with solid notions of procreation as a “natural” process that technologies should not surpass, sometimes aiming to mirror the biological limits of female bodies (Büchler and Parizer 2017). These childbearing norms are notably evident in media coverage, often negatively portraying postmenopausal mothers (Cutas 2007). However, in Europe, perceptions of age deadlines for childbearing have shifted towards later cutoffs (Lazzari, Compans, and Beaujouan 2024). What was deemed “late” in the early 1980s does not refer to the same ages today. In contexts of increasing secularization and cultural individualization, the pursuit of enhanced reproductive autonomy may also drive the adoption of permissive age-based regulations (Büchler and Parizer 2017).

Finally, pronatalist objectives motivate permissive age rules in some contexts, with policies that aim to expand MAR availability to a broad population to boost fertility levels (Büchler and Parizer 2017).

Having presented the factors influencing age-based MAR policies, it is then important to recognize that regulatory frameworks play a crucial role in the contribution of MAR to late births. This is suggested by the fact that the contribution of MAR to births varies between countries with similar degrees of birth postponement but different types of MAR regulation, such as in Southern Europe (Devolder and Borisova 2022). As suggested by previous research, regulations alone are not a sufficient explanatory factor of MAR activity as normative aspects may also shape this influence (Präg and Mills 2017b).

### Normative contexts

Despite the pivotal role of access regulations in comprehending the influence of MAR on births, other factors come into play. For instance, Präg and Mills (2017b) showed that cross-country differences in MAR usage are significantly associated with the degree to which MAR use is socially accepted. Similar to other fertility-related policies, the impact of MAR and its regulation on births may depend on prevailing norms within a context (Neyer, Lappegård, and Vignoli 2013). Within the domain of assisted reproduction and age-based access regulations, individuals may have to cover infertility treatments with out-of-pocket expenses, notably when they are older than the age cutoffs for reimbursement eligibility. Studies indicate that the willingness to pay for infertility treatments, which is the maximum amount of money people are ready to cover to have a child, varies across cultural contexts (Fauser et al. 2019; Skedgel et al. 2021). Institutional factors, such as private social insurance and the prevalence of private clinics over public ones, may also shape the availability of treatments in a country. Possibly, another option for prospective parents is to seek cross-border reproductive care, which comes with additional expenses (Salama et al. 2018; Shenfield et al. 2010).

For these reasons, this paper explores to what extent the relationship between MAR policies and late births can be better understood by also comparing some countries’ normative features. Including such factors in the analysis notably helps to understand differences in the increase in very late births across countries with similar MAR regulations.

### Research questions

The rise in late births has rarely been reported as a strong motivation for implementing permissive age-based regulations (Büchler and Parizer 2017; Martani et al. 2022; Piek, Pennings, and Provoost 2023; Griessler et al. 2022), allegedly because restrictions may have been established when late births were less common than today. However, actual births and intentions to have a child at advanced reproductive ages have become more common in high-income countries (Beaujouan and Sobotka 2017) and perceived upper age limits for motherhood have shifted towards later ages over time (Lazzari, Compans, and Beaujouan 2024). Therefore, this social change regarding shifts in both perceptions and behaviors is likely to challenge strict MAR policies, as removing barriers to people’s desires to have a child may be an increasingly relevant argument in favor of more permissive rules (Zweifel et al. 2020). I first distinguish between various age-based policy responses to late fertility trends in high-income countries. Limitations to public funding are an important policy tool to restrict MAR availability (Chambers et al. 2014). Hence, my first research question asks whether age limitations for the reimbursement of IVF have aligned with, anticipated, adapted to, or lagged behind the increase in births at advanced maternal ages (40+). Distinguishing between different groups of countries based on these features also provides insights into what may have motivated the adoption of specific age cutoffs.

From a demographical point of view, it is also crucial to understand the extent to which MAR techniques may facilitate the recuperation of delayed births at late ages. As a second part of this research, I focus on births occurring from the maternal age of 45, which are unlikely to occur without medical help. My second research question investigates whether countries with the same reimbursement policy responses to late fertility trends have witnessed similar contributions of births at 45+ to births at 40+. In cases when the rise in these very late births varied across countries with comparable age-based reimbursement policies, I explore the regulations of other areas—such as access age-based rules, OD, or POC—and country differences in childbearing norms.

## Data and analytical approach

### Data

#### Age-based regulations for assisted reproduction data (2023)

Information on MAR regulations used in this paper refers to the age-based regulations to access homologous IVF treatments (i.e., without gamete donation) based on women’s and men’s age, the public coverage of IVF treatments (partial or complete, age-based restrictions, number of IVF cycles and birth parity covered) and the regulation of OD and POC (whether OD is allowed and reimbursed, POC regulated and allowed).^2^ The data include the content of enacted rules and the year of adoption of age-based rules and reimbursement schemes. They were gathered by combining various sources, including legal texts or country-specific publications, cross-country comparisons (Calhaz-Jorge et al. 2020; Berg Brigham, Cadier, and Chevreul 2013) such as the International Federation of Fertility Societies Surveillance reports (IFFS 2019, 2022), and the International Reproduction Policy Database (https://irpd.wzb.eu/). All sources were cross-checked with each other. Details on the rules, contradictions found in the literature, and sources used for each country are provided in the Supporting Information (Table A1).

The analysis focuses on high-income countries with public reimbursement schemes for infertility treatments. This leads to the exclusion of Switzerland and Ireland where MAR utilization is not publicly covered. I also exclude countries that never had legal provisions at the national level, like the United States and Canada. Italy is also not included as this country has a direct legal age-based provision at the national level, but regions apply their own rules for reimbursement eligibility. Furthermore, I exclude countries where age limitations for public coverage are not advocated by law, that is, the Netherlands, Norway, and the United Kingdom. In these countries, this is addressed through guidelines for practitioners. The study relies on the timing of age-based regulations for 18 countries for which data were collected exhaustively and nine countries for which information on age restrictions and the timing of public funding is partial.

High-income countries started using IVF at different periods and followed diverse trajectories in regulating and publicly subsidizing treatment usage (Figure 1). Most countries do not have any direct age limit to access treatments, only indirect ones via eligibility to its reimbursement and based on women’s age. Access rules rarely rely on men’s age (Online Table A1). Indirect restrictions are between the female age of 35 and 50 years old. Beyond these specific cutoffs and without a direct age-based limitation, women can undergo MAR interventions with out-of-pocket expenses. Among the countries with direct age limits to access medical help, cutoffs are higher, ranging between 45 and 54 years old (Online Table A1). Some countries also limit the number of reimbursed IVF cycles (between two and seven), and a handful have criteria based on couples’ or women’s birth parity (Online Table A1).^3^

Today, most countries allow OD but do not cover its usage (Table 1). Embryo donation is less often permitted (Calhaz-Jorge et al. 2020), which is why it is not included in the analysis. POC is explicitly not allowed in a few countries (Lithuania, Hungary, Austria) and is not ruled out in many countries (Table 1).

#### Late and very late fertility

**FIGURE 2:**
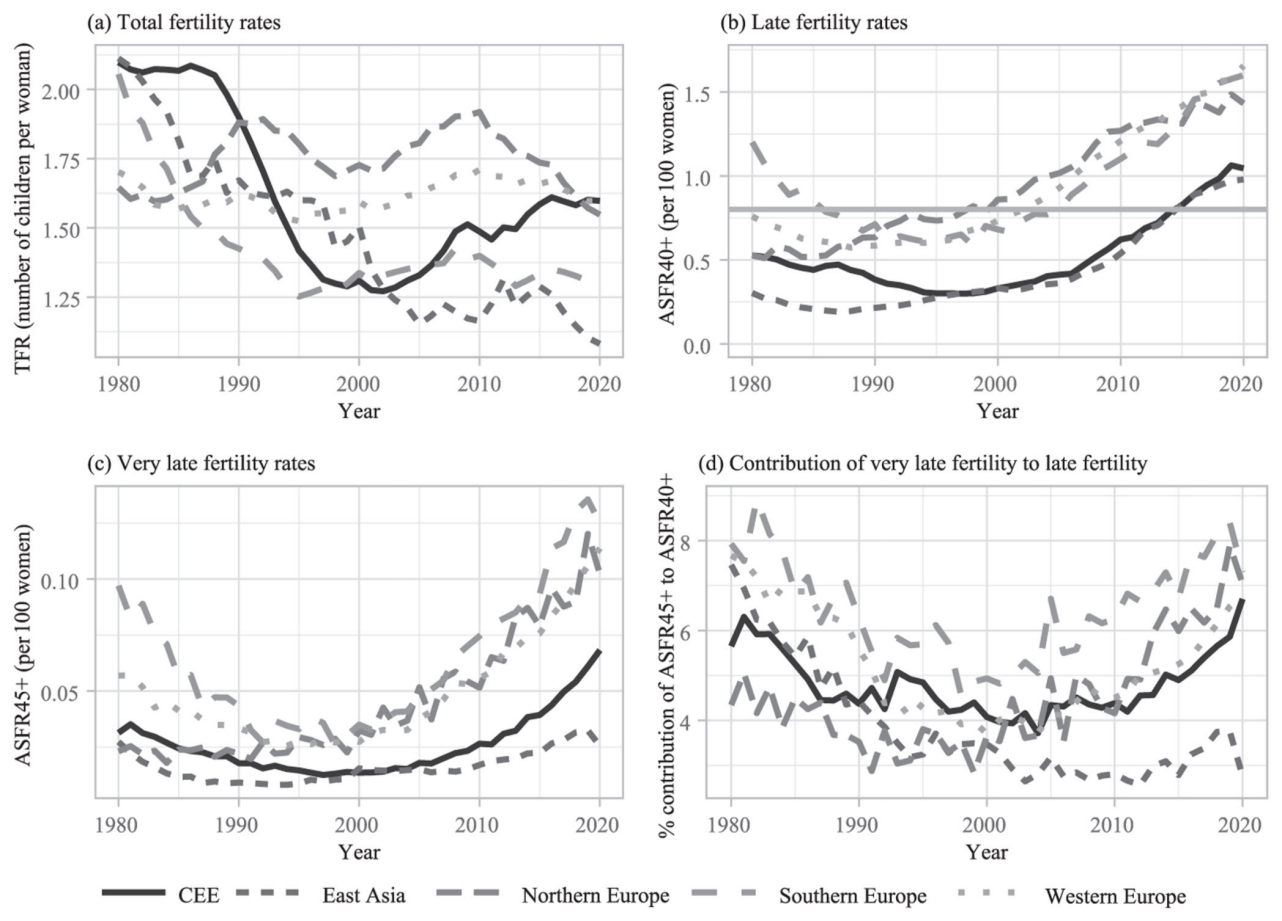
Trends in (very) late fertility by region of low-fertility countries (1980–1920) SOURCE: Human Fertility Database (2023) and Eurostat (2023).

Data on fertility are retrieved from the Human Fertility Database (https://www.humanfertility.org/) and Eurostat (https://ec.europa.eu/eurostat). Figure 2 shows various fertility indicators by region.^4^ All analyzed countries exhibit low total fertility rates (TFR) since the 1990s, that is, below 2.1 children per woman (Figure 2a). Late fertility is considered at the maternal age of 40 or over (Figure 2b). Very late fertility refers to births from women aged 45 or over (Figure 2c). Age-specific fertility rates (ASFR40+ and ASFR45+) are computed up to age 49 (Figure 2d). They refer to the average number of children per 100 women in the corresponding age groups (40–49 and 45–49).

From 1980 to 2020 in the analyzed countries, there have been between 0.2 and 1.7 children per 100 women aged 40+. Given this range, levels of late fertility appear relatively low in Central and Eastern European countries (CEE) and Eastern Asian countries until the mid-2010s. They started increasing later in Southern European countries compared to Northern Europe (from the late 1990s vs. 1980s to early 1990s) but with rapidly growing late birth rates. This seems partly driven by a rise in very late births since the 2000s (Figure 2c). ASFR45+ has also increased in other regions, except East Asia.

#### Aggregated indicators of attitudes towards childbearing

The analysis also includes differences in aggregated attitudes towards childbearing, considered proxies for prevailing norms at a country level, by utilizing harmonized surveys for several European contexts. The analysis of such indicators is exploratory but provides potential explanations for differences in the degree of permissiveness of age-based regulations (first part of the analysis) and in the rise of births from age 45 across countries with similar age-based reimbursement regulations (second part of the analysis).

The perceived upper age limits for childbearing at the country level are calculated with rounds 3 (2006–2007) and 9 (2018–2019) of the European Social Survey. This indicator can be illustrative of whether age norms align with the age-based regulations and together contribute to limiting or enhancing very late fertility. This indicator relies on a question about the age at which a woman is considered too old to conceive. Respondents could also say that they do not have any opinion (“don’t know”) or that it is “never too old” to conceive. Although shares of acknowledgment of a specific age are high—between 87 percent and 99 percent depending on the country and survey year (Lazzari, Compans, and Beaujouan 2024)—cross-country differences in such shares can reflect variations in the strength of age norms for childbearing. Therefore, I adjust crude average values by shares of acknowledgment of an opinion and rely on adjusted upper age limits displayed in Table 2.^5^ Most countries exhibit an average above the age of 40.

I also include a measure of the social importance of parenthood with cross-sectional data from the European Values Study (EVS), using waves 4 (2008–2010) and 5 (2017–2020). This is based on the aggregated proportion of respondents within a country who (strongly) agreed with the fact that “it is a duty towards society to have children”—versus (strongly) disagreeing or not acknowledging an opinion. This indicator ranges from 3 percent to 77 percent and is lower in Northern and Western European countries than in CEE and Southern Europe (Table 2). This attitudinal measurement can indicate the importance of becoming a parent within a society. It is therefore relevant to understand the nonalignment between age-based MAR policies and late fertility levels and to which extent individuals may be willing to pay for IVF treatments when facing difficulties in having children in contexts where strict age cutoffs for reimbursement eligibility apply.

### Method: Distinguishing between countries’ age-based reimbursement policy responses to late fertility trends

To answer the first research question, I develop a typology of distinct countries’ policy responses to late fertility trends. Existing cross-comparison of age-based MAR policies often focus on current rules, failing to capture changes (Berg Brigham, Cadier, and Chevreul 2013; Büchler and Parizer 2017; Calhaz-Jorge et al. 2020). Existing categorizations of countries have also combined information on both rules that govern MAR access in general and regulations that determine eligibility for public reimbursement (Büchler and Parizer 2017). However, these two restrictions may stem from different motivations. In addition, many countries have only adopted indirect age-based restrictions and do not apply any additional cutoffs for MAR access at one’s own cost (Berg Brigham, Cadier, and Chevreul 2013; Calhaz-Jorge et al. 2020). The typology presented in this paper distinguishes between countries based on (1) the degree of permissiveness of age-based reimbursement policies and (2) the rates of late birth from the maternal age of 40 (late fertility) when such policies were adopted and revised. Given the range of applied age-based restrictions for reimbursement eligibility in the analyzed countries (cf. the Age-Based Regulations for Assisted Reproduction Data (2023) section), I consider age-based criteria for reimbursement as strict when cutoffs are 42 years old or below, and as permissive when higher or when there is no limitation.^6^ Late birth rates are relatively high when ASFR40+ surpasses 0.8 children per 100 women, moderate when close to this value, and low when below (horizontal gray line in Figure 2b; cf. the Late and Very Late Fertility section). Based on whether age-based regulations for reimbursement are strict or permissive, and whether late fertility levels were high or low when rules were adopted, I descriptively identify six types of countries which are detailed in the Result section (Table 3).

The second research question examines the contribution of fertility rates at age 45 or over to fertility rates at age 40. Estimates of reproductive capacities typically stop at 45 (Leridon 2004) as without any medical help, the probability of being permanently sterile is close to 100 percent above this age (Leridon 2008). In line with this, births among women over 45 have shown a moderate increase in Europe since the 1980s (Billari et al. 2007). Yet, the evolution of assisted reproduction techniques might enhance this trend (Beaujouan 2020). Given that births over the female age of 45 are more unlikely to occur without any medical intervention, an increasing contribution of very late fertility to late fertility may indicate where MAR has a relevant impact on births. I identify the regulatory framework in which this has been observed. In addition, examining childbearing attitudes aggregated at the country level provides insights into within-group differences.

## Results

### Age-based policy responses to late fertility trends

**FIGURE 3:**
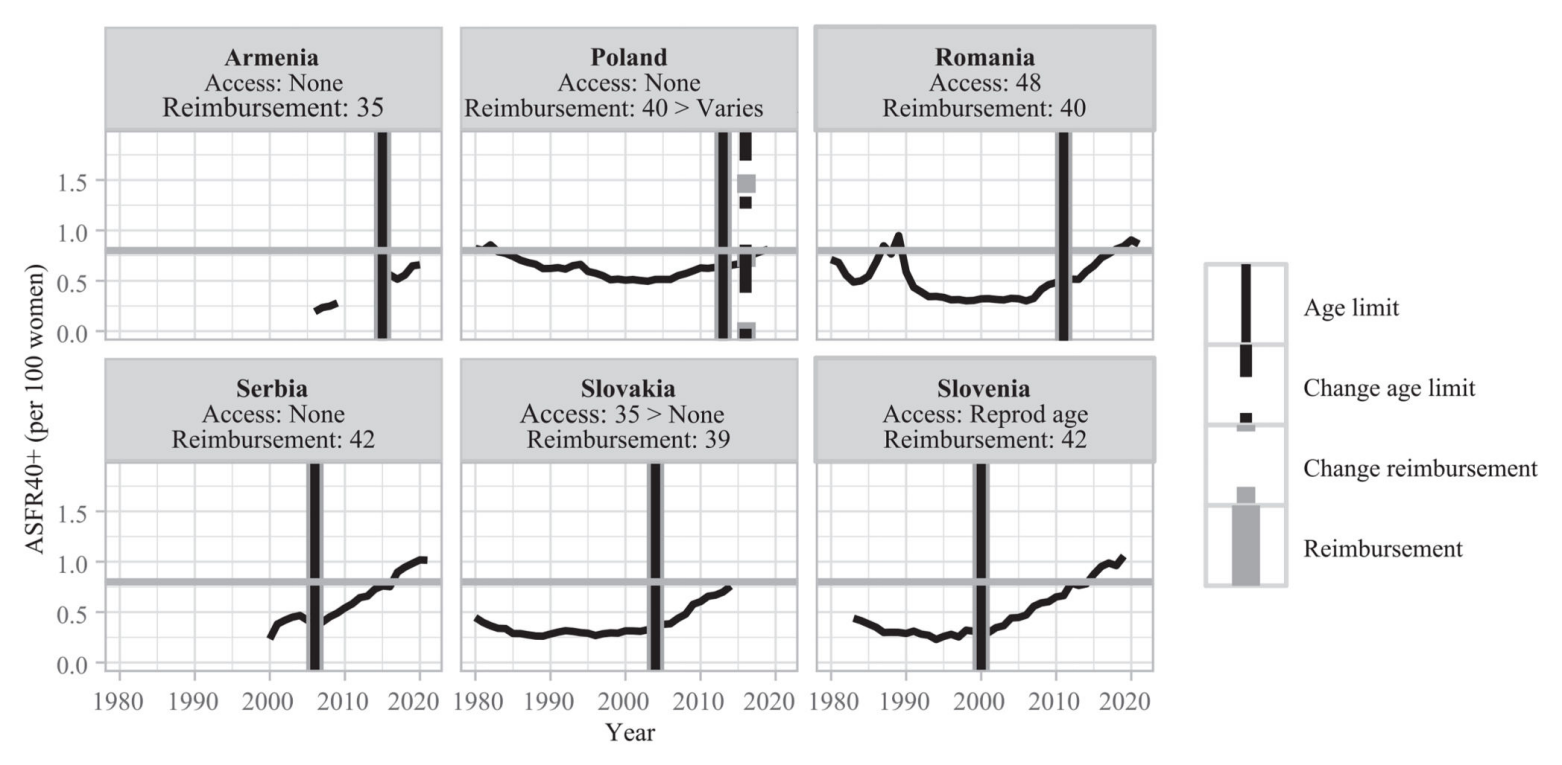
Trends in ASFR40+, years of legislation (vertical bars), and age limit for access and public coverage—countries with restrictive age rules for IVF reimbursement and low late fertility rates NOTE: Trends in ASFR40+ are plotted as black solid lines. The horizontal gray line indicates the threshold from which ASFR40+ is considered as “high.” Solid vertical lines correspond to the year of adoption of reimbursement schemes for homologous IVF treatment (in gray) and of an age limit to be eligible for reimbursement (in black). Dashed/dotted vertical lines indicate when a change occurred (in gray for reimbursement schemes and in black for age-based rules). Age thresholds for reimbursement and access are indicated below the country names. When a specific age cap or a change for reimbursement eligibility is indicated without any vertical line, the information on legislation timing is missing. SOURCE: Human Fertility Database (2023), Eurostat (2023), and the Age-based regulations for assisted reproduction data (2023); see the Supporting Information.

**FIGURE 4:**
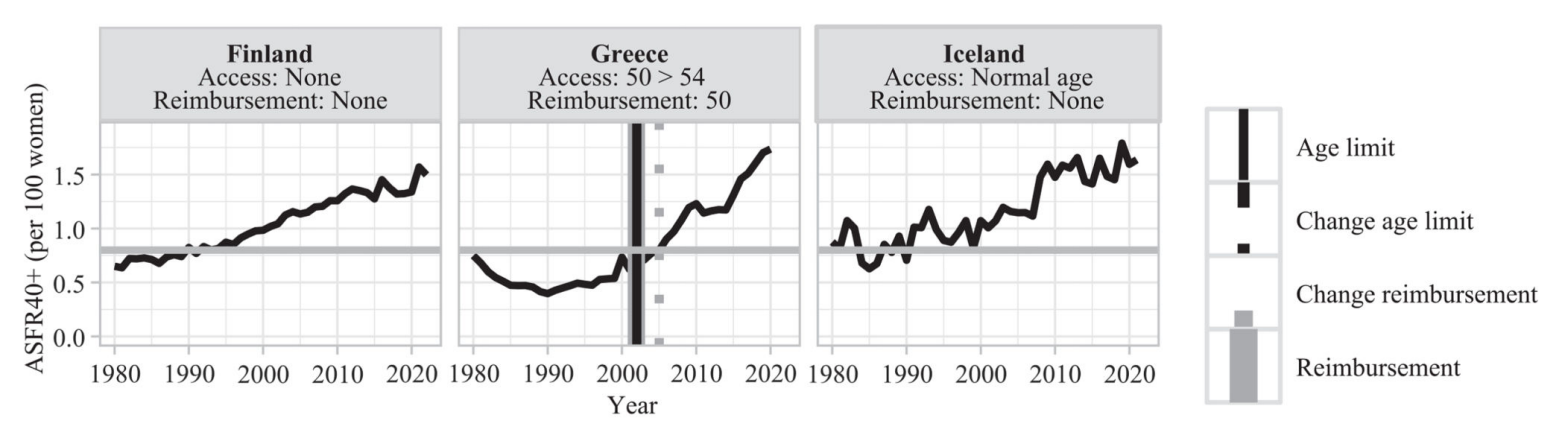
Trends in ASFR40+, years of legislation (vertical bars), and age limit for access and public coverage—countries with permissive age rules and high late fertility rates NOTE: Trends in ASFR40+ are plotted as black solid lines. The horizontal gray line indicates the threshold from which ASFR40+ is considered as “high.” Solid vertical lines correspond to the year of adoption of reimbursement schemes for homologous IVF treatment (in gray) and of an age limit to be eligible for reimbursement (in black). Dashed/dotted vertical lines indicate when a change occurred (in gray for reimbursement schemes and in black for age-based rules). Age thresholds for reimbursement and access are indicated below the country names. When a specific age cap or a change for reimbursement eligibility is indicated without any vertical line, the information on legislation timing is missing. SOURCE: Human Fertility Database (2023), Eurostat (2023), and the Age-based regulations for assisted reproduction data (2023); see the Supporting Information.

**FIGURE 5:**
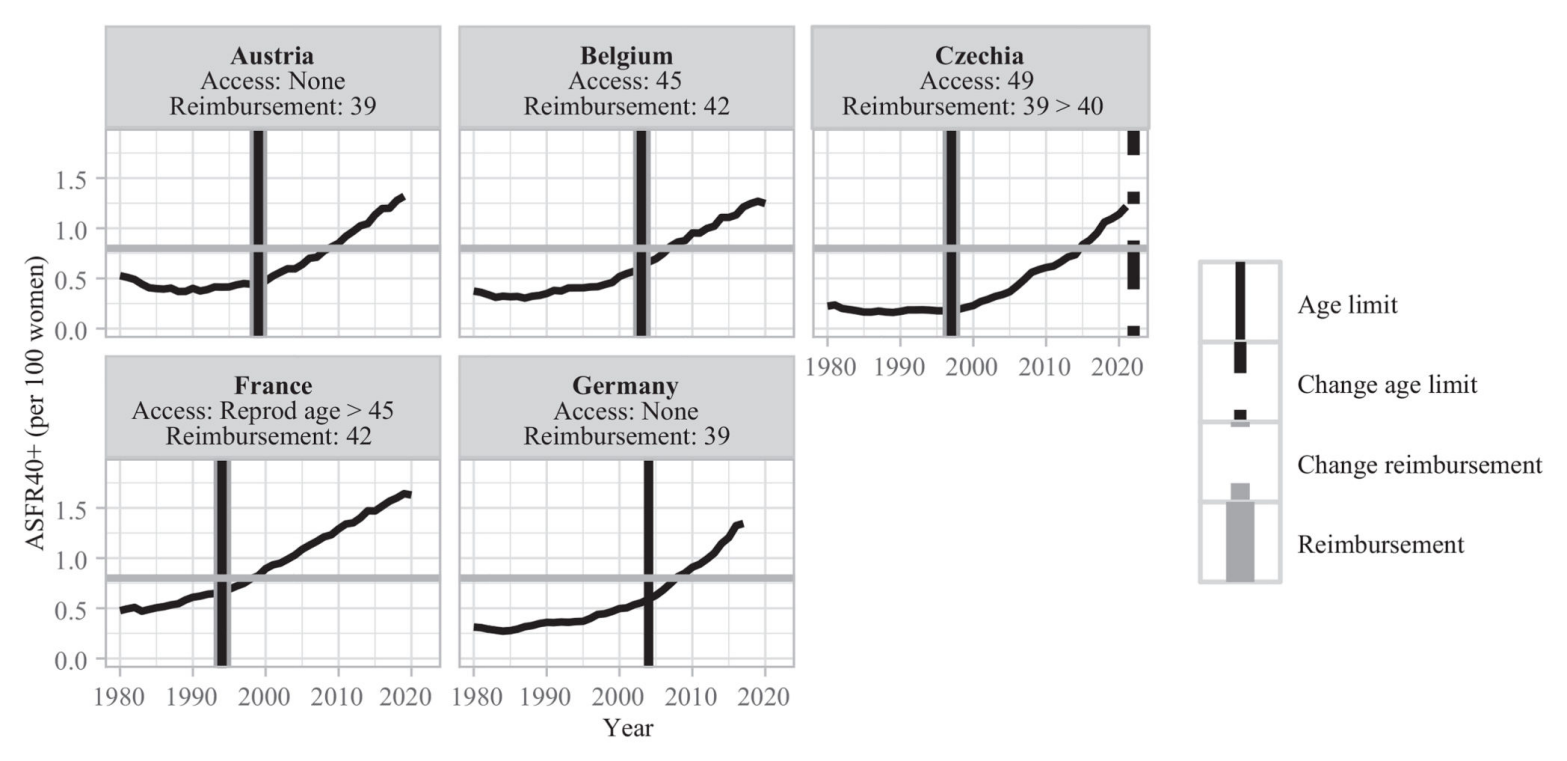
Trends in ASFR40+, years of legislation (vertical bars), and age limit for access and public coverage—countries with low late fertility rates when restrictive age rules for IVF reimbursement were adopted and no shift towards more permissiveness NOTE: Trends in ASFR40 + are plotted as black solid lines. The horizontal gray line indicates the threshold from which ASFR40+ is considered as “high.” Solid vertical lines correspond to the year of adoption of reimbursement schemes for homologous IVF treatment (in gray) and of an age limit to be eligible for reimbursement (in black). Dashed/dotted vertical lines indicate when a change occurred (in gray for reimbursement schemes and in black for age-based rules). Age thresholds for reimbursement and access are indicated below the country names. When a specific age cap or a change for reimbursement eligibility is indicated without any vertical line, the information on legislation timing is missing. SOURCE: Human Fertility Database (2023), Eurostat (2023), and the Age-based regulations for assisted reproduction data (2023); see the Supporting Information.

**FIGURE 6:**
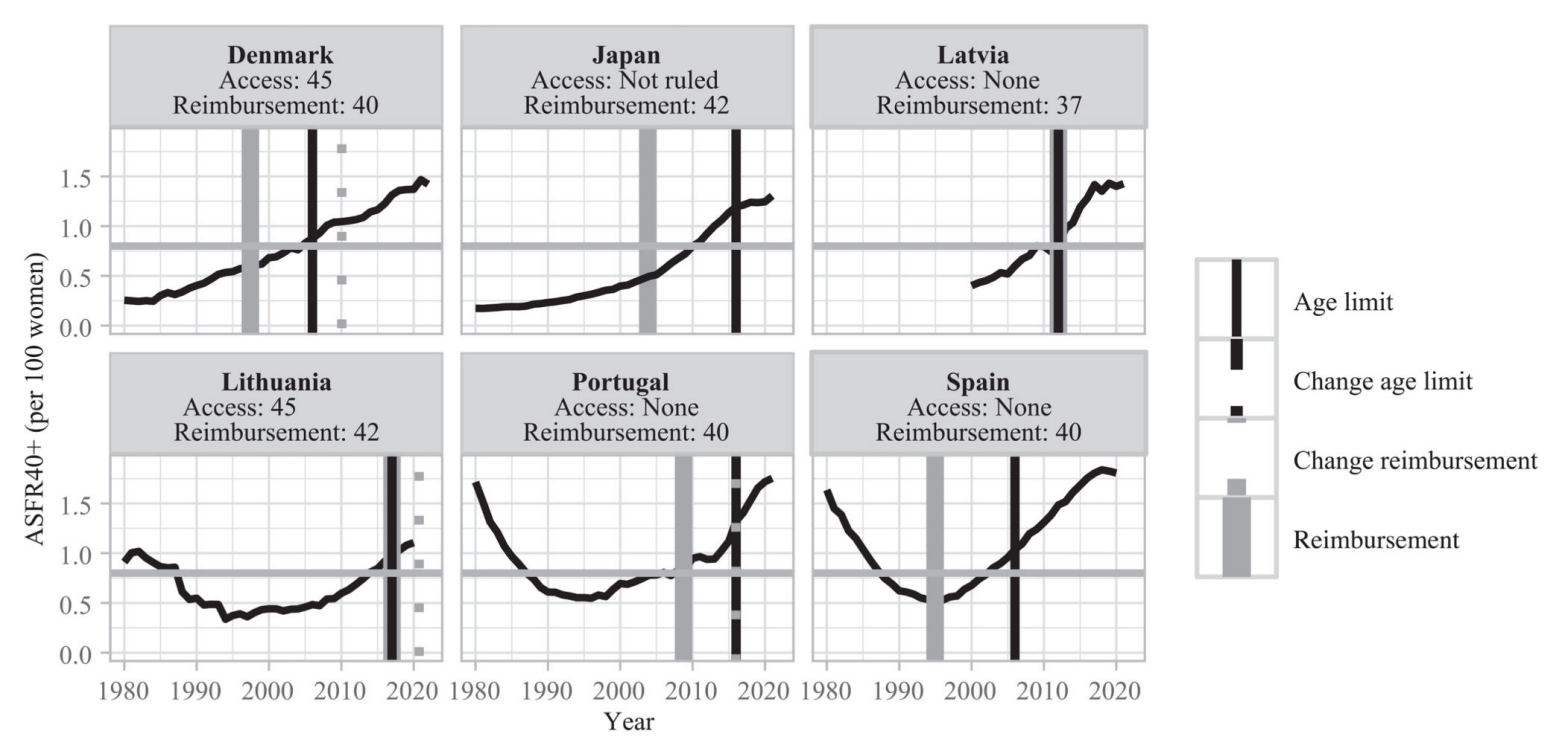
Trends in ASFR40+, years of legislation (vertical bars), and age limit for access and public coverage—countries with restrictive age rules and high late fertility rates without any law adaptation NOTE: Trends in ASFR40+ are plotted as black solid lines. The horizontal gray line indicates the threshold from which ASFR40+ is considered as “high”. Solid vertical lines correspond to the year of adoption of reimbursement schemes for homologous IVF treatment (in gray) and of an age limit to be eligible for reimbursement (in black). Dashed/dotted vertical lines indicate when a change occurred (in gray for reimbursement schemes and in black for age-based rules). Age thresholds for reimbursement and access are indicated below the country names. When a specific age cap or a change for reimbursement eligibility is indicated without any vertical line, the information on legislation timing is missing. SOURCE: Human Fertility Database (2023), Eurostat (2023), and the Age-based regulations for assisted reproduction data (2023); see the Supporting Information.

**FIGURE 7:**
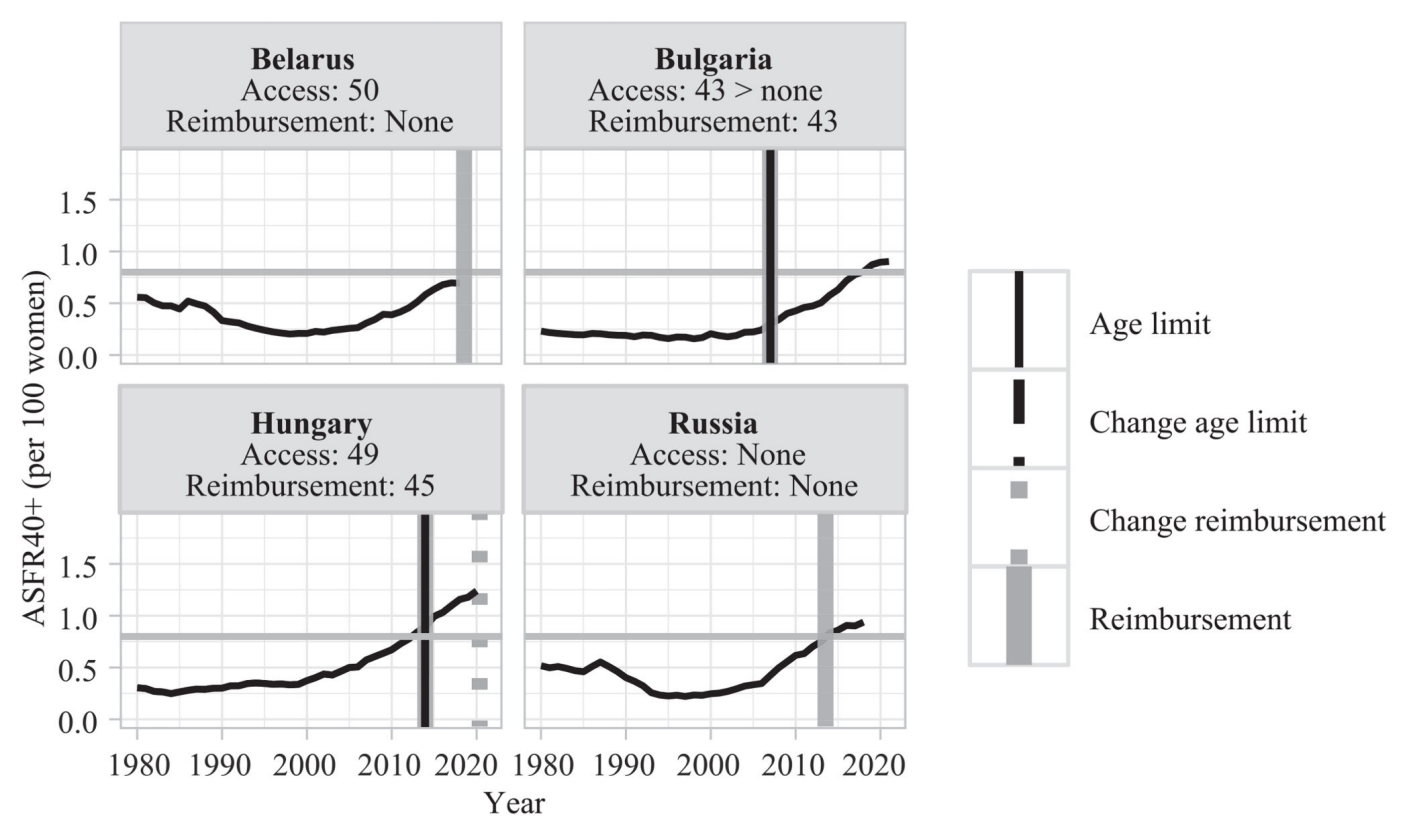
Trends in ASFR40+, years of legislation (vertical bars), and age limit for access and public coverage—countries with permissive age rules and low/moderate late fertility rates NOTE: Trends in ASFR40 +are plotted as black solid lines. The horizontal gray line indicates the threshold from which ASFR40+is considered as “high.” Solid vertical lines correspond to the year of adoption of reimbursement schemes for homologous IVF treatment (in gray) and of an age limit to be eligible for reimbursement (in black). Dashed/dotted vertical lines indicate when a change occurred (in gray for reimbursement schemes and in black for age-based rules). Age thresholds for reimbursement and access are indicated below the country names. When a specific age cap or a change for reimbursement eligibility is indicated without any vertical line, the information on legislation timing is missing. SOURCE: Human Fertility Database (2023), Eurostat (2023), and the Age-based regulations for assisted reproduction data (2023); see the Supporting Information.

**FIGURE 8:**
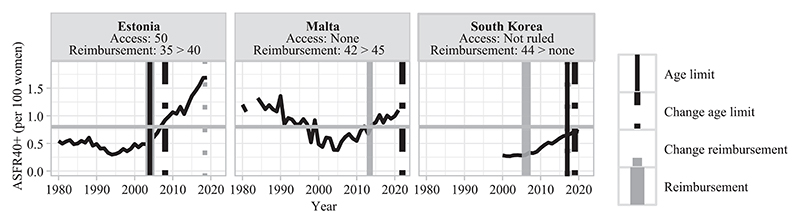
Trends in ASFR40+, years of legislation (vertical bars), and age limit for access and public coverage—adaptations of age rules to the increase in late fertility rates NOTE: Trends in ASFR40+ are plotted as black solid lines. The horizontal gray line indicates the threshold from which ASFR40+ is considered as “high.” Solid vertical lines correspond to the year of adoption of reimbursement schemes for homologous IVF treatment (in gray) and of an age limit to be eligible for reimbursement (in black). Dashed/dotted vertical lines indicate when a change occurred (in gray for reimbursement schemes and in black for age-based rules). Age thresholds for reimbursement and access are indicated below the country names. When a specific age cap or a change for reimbursement eligibility is indicated without any vertical line, the information on legislation timing is missing. SOURCE: Human Fertility Database (2023), Eurostat (2023), and the Age-based regulations for assisted reproduction data (2023); see the Supporting Information.

This section describes different types of countries based on the late fertility rates observed at the time of adoption of age-based eligibility criteria for treatment reimbursement (Table 3). In Figures 3–8, fertility trends are plotted from the 1980s to 2020s, with ASFR40+ as solid black lines. Solid vertical lines display the year of adoption of reimbursement schemes for IVF (in gray) and age-based rules to benefit from them (in black). The corresponding age cutoffs for reimbursement are displayed, along with age-based regulations for accessing IVF (although these latter aspects did not determine the grouping of countries). I provide information on the extent of public coverage (partial or complete and the number of reimbursed IVF cycles) in the text and Online Table A1. The dotted and dashed lines indicate the years of regulation changes.

#### Restrictive age rules and relatively low rates of late fertility from the 1980s onwards (alignment)

The first group of countries are CEE contexts with strict age-based policies to access public funding (age 42 or below) that are in line with low levels of late fertility at the time of law adoption (below 0.8 children per 100 women).^7^ Although late fertility rates have been on the rise since the 1990s, they remain relatively low and only recently reached high levels comparable to those of, for instance, Western European countries (Figure 3). These restrictive age cutoffs also align with the average perceived age deadline for motherhood, which was below age 40 in these countries (except for Slovenia, cf. Table 2).

#### Permissive age rules and high late fertility rates (alignment)

Countries of the second group have adopted permissive age-based regulations for reimbursement (no age restriction or at the late age of 50) that align with high late fertility rates. In Greece, these reimbursement schemes were adopted in 2002 and were increased to full coverage in 2005 (Figure 4). In Iceland and Finland, reimbursement schemes have been available since the beginning of MAR medical practice without any age limitation for eligibility (Granberg, Wikland, and Hamberger 1998). Direct age regulations are also permissive, and reasons for this high degree of permissiveness may be multifaceted and vary by country.^8^

#### No adaptation of restrictive indirect age-based rules which were adopted when late fertility rates were low (lagging behind)

Countries of the third group adopted strict age-based eligibility criteria for treatment reimbursement in the 1990s–2000s when late birth rates were low, but are lagging behind subsequent upward trends in late fertility (Figure 5). Age limits of 42 years old or below were consistent with fertility levels at that time but have not been revised since then despite significant increases in late births (or only marginally adjusted, with a one-year increase in Czechia in 2022). In these countries with broad treatment coverage for most of them, the social cost of MAR interventions may be one of the main motivations behind maintaining these regulations.^9^

#### Restrictive age-based rules to access public coverage adopted when late fertility rates were high and no adaptation since then (lagging behind)

Age-based policies for treatment reimbursement in the fourth group are also lagging behind upward trends in late fertility, but restrictive age rules for reimbursement were adopted when late fertility rates were high. These age-based rules can be explained by the generosity of the security health systems and the will to limit the social cost of treatments.^10^ This motivation is notably indicated by the lag between the implementation of reimbursement schemes and the adoption of age-based criteria for eligibility, as in Denmark, Spain, Portugal, and Japan (Figure 6).

#### Permissive age-based rules and low/moderate levels of late fertility (anticipation)

The fifth group comprises four CEE countries that implemented reimbursement schemes for infertility treatments with permissive age-based rules, despite low or moderate late fertility rates at the time of regulation adoption. These regulations can be seen as anticipating subsequent upward trends in late fertility (Figure 7). Russia and Belarus have no age limit for reimbursement eligibility, while Hungary adopted a cutoff of 45. Direct age rules to access IVF are also rather permissive, as countries of this group have no specific age cutoff or at old ages (50 years old in Belarus). Their permissiveness can be explained by pronatalist motivations to allow more couples to access MAR and have a child (Rusanova 2020; Szalma and Bitó 2021),^11^ in contexts where many people believe that parenthood is a duty (36–77 percent, depending on the country of this group; Table 2).

#### Revised age-based regulations along upward late fertility trends (adaptation)

In countries of the last group (Malta, Estonia, and South Korea), age regulations regarding public reimbursement have been revised consistently with upward late fertility trends. Like for the previous group, such adaptation of rules can be motivated by pronatalist aims (Grant et al. 2006; Kim 2019), as these three countries have also increased the number of publicly covered IVF cycles. For instance, Estonia changed the three-cycle limit for unlimited reimbursed IVF cycles in 2008 and the public coverage has been extended from partial to complete in 2018. In South Korea, the number of covered IVF cycles also increased from six to seven in 2019.

### Age-based regulations and very late fertility

#### Very late fertility trends by type of age-based regulation for IVF reimbursement

**FIGURE 9:**
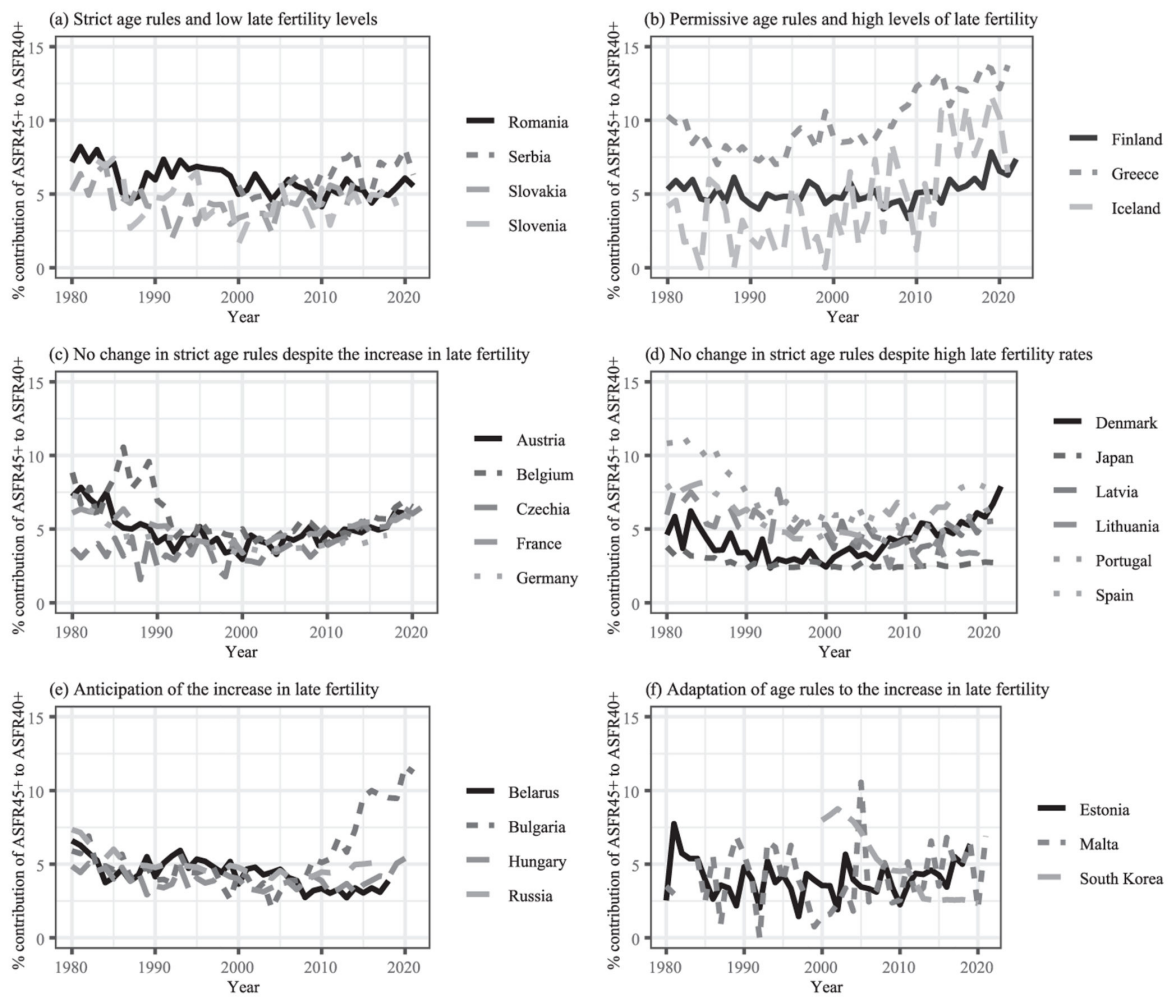
The contribution of ASFR45+ to ASFR40+ by type of countries’ age-based policy response to late fertility trends SOURCE: Human Fertility Database (2023), Eurostat (2023), and the Age-based regulations for assisted reproduction data (2023); see the Supporting Information.

Based on the different groups of countries described above, this section now explores whether countries with similar age-based reimbursement policy responses to late fertility trends witnessed similar evolutions in very late births that are unlikely to occur without medical help. To address this second research question, Figure 9 displays trends in the contribution of ASFR45+ to ASFR40+ among countries of each group.

Among the first group of aligned strict age-based rules for treatment reimbursement with low late fertility rates, the contribution of very late births to late births fluctuated between 3 percent and 7 percent (Figure 9a). This contribution remained rather stable over time, except for Serbia where it has particularly increased since 2010.^12^

For countries with aligned permissive age-based regulations with high levels of late fertility (Figure 9b), the contribution of very late births to late births particularly rose in Greece (it increased between 2005 and 2021, from 9 percent to 14 percent) and in Iceland since 2010 (reaching 10 percent in 2020) but slightly in Finland (from 5 percent in 1980 to 7 percent in 2022).

For contexts where age-based reimbursement policies are lagging behind ASFR40+ trends, the contribution of ASFR45+ to ASFR40+ increased since the mid-1990s, similarly across countries of this group (Figure 9c). It is a bit lower in Germany (below 5 percent in the late 2010s vs. above 5 percent in the other countries), where IVF treatments below age 40 are partially funded.

Conversely, the contribution of ASFR45+ to ASFR40+ greatly differs between countries where age-based policies for reimbursement are also lagging behind late fertility trends but were adopted later than in the previous group (Figure 9d). From the mid-2000s, it particularly rose in Spain (from 5 percent before 2005 to 8 percent in 2020) and Denmark (from less than 3 percent to 5 percent). This is observed despite a direct upper age restriction of 45 years old in Denmark while Spain has none.

In countries with anticipatory age-based reimbursement regulations, the contribution of ASFR45+ to ASFR40+ rose after reimbursement schemes were implemented, particularly in Bulgaria (from 5 percent in 2010 to 12 percent in 2020). The slight increase in the ASFR45+ contribution to ASFR40+ in Hungary (from 3 percent in 2011 to 5 percent in 2020) is observed despite a 45-age limit to be eligible for reimbursement (Figure 9e).

In the last group of countries where age-based reimbursement policies have adapted to late fertility trends, the limited contribution of ASFR45+ to ASFR40+ has remained constant and even decreased in South Korea (Figure 9f).

#### Possible explanations for the variation in very late fertility trends among countries with similar age criteria for IVF

Explanations for discrepancies within each group can regard differences in childbearing norms as well as other regulated MAR areas such as direct age-based regulations (which can restrict women from accessing infertility treatments with out-of-pocket money) and the regulation of OD and POC (which have the potential to compensate for the natural decline of fecundity with age).

In the first group of countries, strict access age restrictions seem to limit the impact MAR may have on births in women’s late forties. Indeed, in Serbia, where very late fertility has particularly risen (Figure 9a), women cannot benefit from the full public coverage of IVF above age 42 but private health insurance can cover treatment use (Čunderlík Čerbová 2022) with-out any age-based limitation (Figure 3). In contrast, there is a direct age limit to access IVF in the other countries of this group, which can explain their lower contributions of ASFR45+ to ASFR40+. Those age limits are 48 with a woman’s own eggs and 50 with donated eggs in Romania.^13^

Such explanation does not seem to explain differences in very late fertility trends across the countries where permissive age-based regulations for IVF reimbursement align with high levels of late fertility (Figure 9b). There is no specific age limit to access IVF in Finland, at a late age in Greece (increased from 50 to 54), and a soft one in Iceland (restricting access to individuals of “normal childbearing age” since 1996). OD is legally permitted in these three countries. POC is practiced but not ruled in Iceland and Finland and has been allowed since 2022 in Greece. Perceptions of upper age limits for motherhood and for parenthood as a social duty are similar in both Northern European countries (Table 2, no data for Greece). Possibly, the demand for medical help may also be similar in these contexts, as late fertility rates are as high and similarly on the rise (Figure 4). However, recent research reported that, in practice, access to IVF treatments in public clinics is limited to women in their forties in Finland (Calhaz-Jorge et al. 2020), which could explain the limited rise in the contribution of ASFR45+ to ASFR40+ in this country. Given the permissiveness of legislation on infertility treatments in this group, whether MAR contributes to late fertility may greatly depend on differences in medical practice from one country to another.

In the third identified group of countries, trends in very late fertility are similar (Figure 9c), and this is observed despite differences in age-based limitations to access IVF with or without co-payment and out-of-pocket money,^14^ and in the timing of OD legislation.^15^ Average perceived age deadlines for motherhood are also high (above age 40), except for Czechia (Table 2). In this group of countries, age-based limitations for treatment reimbursement may be sufficient to explain the limited increase in very late fertility over time.

Different factors may explain the variation in ASFR45+ trends within the other group of countries where age-based regulations for IVF reimbursement are lagging behind upward trends in late fertility (Figure 9d). Denmark and Spain, where very late births have particularly risen, both rank high regarding MAR activity and OD use in Europe (Smeenk et al. 2023). To a lesser extent, the contribution of very late fertility to late fertility has also risen in Latvia and Portugal since the adoption of public coverage in the mid-2010s, although with a 39-year-old age limit for treatment reimbursements (Figure 6). In both countries where, respectively, 29 percent and 40 percent of EVS respondents in 2008–2010 believed that parenthood is a social duty (Table 2) and access with out-of-pocket money is not limited by age (Figure 6), this increase in very late births may be explained by the willingness to pay for treatments above the reimbursement age restriction. Conversely, the ASFR45+ contribution to ASFR40+ has remained constant in Japan and even slightly decreased in Lithuania before remaining constant since the mid-2000s. In this latter context, direct access age restrictions may limit the contribution of births at 45+ to births at 40+.

In countries with pronatalist policy goals with anticipatory policy responses to fertility trends, parenthood is highly valued (Table 2), and therefore willingness to pay for IVF procedures may explain part of the increase in very late births despite age restrictions for reimbursement eligibility. In addition, in Bulgaria, the significant increase in the contribution of ASFR45+ to ASFR40+ was concomitant with the removal of a direct age limit to MAR use in 2011, despite a 43-age restriction to treatment public funding (Figure 7).

Finally, for countries with adapting policies to late fertility trends, differences in the contribution of very late births to late births are not explained by direct age-based policies as they are permissive in the three contexts (Figure 8). Information on childbearing norms is lacking. Possibly, access restrictions other than treatment reimbursement in South Korea may explain the decline in very late births (introduction of a ban on OD and use of preserved frozen eggs only for married women).

## Conclusions and discussion

This paper provides an overview of past and current age-based legislation governing assisted reproduction in various high-income countries, whether it regards direct access to infertility treatments or eligibility for their public reimbursement. It also identifies reimbursement policies’ responses to fertility trends and the regulatory contexts where MAR is likely to influence very late births.

First, the study comprehensively describes different types of policy responses to the increasing late fertility rates observed in high-income countries since the first ART births. In two groups of countries, age rules to be eligible for public reimbursement of infertility treatments aligned well with the prevalence of births at age 40+ over the years, either because rules are restrictive and late fertility rates have remained low (first group) or because regulations were permissive in contexts of high late fertility rates (second group). However, in most high-income contexts, age-based regulations do not align with the current prevalence of late births. For two groups of countries, age-based policies for treatment reimbursement are lagging behind upward trends in late births. Indeed, in the third group, indirect age rules were consistent with late fertility rates at the time of their enactment but have not been revised despite births over the maternal age of 40 increasing sharply. Strong views against permissive indirect regulations may aim to limit the social costs of MAR treatments. This is manifest in the fourth group, which encompasses countries where age-based restrictions were adopted when late fertility rates were already high. Pronatalist motivations may have influenced the MAR regulations of the last two groups, explaining permissive legislation that anticipated the rise in late fertility in the fifth group, and the adapting age-based policies to fertility trends in the last group.

Second, I highlight regulatory frameworks where births at 45+ have particularly increased. This may indicate where MAR may have significantly contributed to increases in late fertility since these births are unlikely to occur without medical help. As a general observation, births at 45+ contributed differently to the overall increase in births at 40+ in contexts with comparable age-based restrictions for reimbursement (except for the third group). For some countries, the absence of any age limits or late cutoffs to access infertility treatments (despite restrictive age-based restrictions for reimbursement) seems to favor births from age 45. Direct access rules may give women more leeway to use MAR at very late ages, even if they have to do so at their own expense. Related to that, what individuals are willing to pay to have a child, through private health insurance or direct out-of-pocket money in domestic private clinics (Fauser et al. 2019) or abroad (Skedgel et al. 2021) may explain differences in ASFR45+. Other determinants of very late birth rates may also be linked to variations in the age profiles of patients de facto accepted in fertility clinics and the regulation and utilization of OD.

One limitation of this work is that it does not assess whether behavioral changes have influenced legislation or vice versa. Instead, it provides an original description of age-based regulations and their relationship with late fertility trends, which brings valuable insights into which contexts are relevant to conduct causal analyses with more comprehensive data. Even though ASFR45+ are indicative of an influence of MAR on fertility, to comprehensively assess the impact of assisted reproduction on actual late birth rates, legislation should be compared with MAR activity and MAR outcomes with or without gamete donation. For this reason, I also do not assess the impact of reproductive technologies on countries’ total fertility levels and the extent to which delayed births are recuperated or foregone. This is a relevant question as some countries promote MAR’s enhanced accessibility as a pronatalist policy tool (Grant et al. 2006; Kim 2019; Rusanova 2020; Szalma and Bitó 2021). Previous work with data from France estimated that the contribution of assisted reproduction is significant before 40 years old, but minor above this age, thus having a limited influence on the TFR (Leridon 2017). The MAR contribution to births over age 40 may be higher in contexts where OD and POC are more used, such as Australia (Lazzari, Gray, and Chambers 2021). At the same time, uncertainty looms regarding the demographic impact of these techniques as the supply of donated eggs does not meet the demand and regarding whether women return to their frozen oocytes (Johnston et al. 2021; Vos and Blockeel 2018). As for now, comparative data over time by age are lacking, particularly on OD and POC. Despite the lack of a causal framework, the analysis presented in this paper suggests that similar age-based restrictions may not always have the same influence on births depending on other regulated areas, past fertility trends, and the normative context in which different combinations of MAR-related policy tools are implemented.

I also solely focus on trends in female fertility and regulations based on women’s age. Late fertility among men has followed the same trend as women (Beaujouan 2020), but only a few countries have adopted age restrictions for men (Online Figure A1).^16^ However, adverse pregnancy outcomes related to the paternal age are increasingly documented (Jimbo et al. 2022). Gender norms have also departed from a “traditional” division of roles, and fatherhood tends to be more associated with childrearing in high-income countries (Braverman 2017). In addition, upper age limits for fatherhood have been increasingly acknowledged by Europeans (Lazzari, Compans, and Beaujouan 2024). Against this backdrop, it is possible that more countries will adopt age limitations to access MAR based on men’s age.

To conclude, this paper shows that restrictive age-based rules to benefit from public support of treatment use do not limit the increase in very late birth rates in some countries like Denmark and Spain, hinting at the role of substantial out-of-pocket contributions. This may influence social disparities in family formation. Differences in MAR use and outcome by socioeconomic groups have been recently shown in several high-income countries (Alon and Pinilla 2021; Dhalwani et al. 2013; Goisis et al. 2023; Groes, Yee, and Iorio 2017; Lazzari and Tierney 2023; Passet-Wittig et al. 2023; Shin et al. 2020). Research should further explore the influence of regulations on inequalities in family formation through MAR as women with more financial resources have greater access to infertility treatments beyond age restrictions for public funding.

Finally, this paper identifies contexts where there is room for potential increased permissiveness in age-based rules, given the nonalignment between late fertility patterns and legislation. Nevertheless, flexible rules alone are no panacea to the challenges associated with delayed births (Botev 2015; Leridon 2017). Trends in birth delay may induce a higher demand for medical help to conceive that does not necessarily correspond with a proportional increase in MAR births (Gleicher, Kushnir, and Barad 2019). Treatment outcomes are also uncertain and can cause psychological distress (Greil et al. 2011). Some scholars also question whether greater MAR availability could adversely influence people’s perceptions of the extent of their reproductive life, leading to a delay in their fertility intentions (Sobotka et al. 2008; Ziebe and Devroey 2008). In turn, whether legislation is permissive or restrictive, it is crucial to accompany policies with effective communication about the limitations of MAR to avoid fueling false hopes.

## Supplementary Material

Supplementary material

## Figures and Tables

**TABLE 1:** Legislation of oocyte donation and planned oocyte preservation by country in 2018–23

Country	Oocyte donation	Oocyte donation is publicly funded	Planned oocyte preservation
Austria	Allowed	No	Not allowed
Armenia	Allowed	No	No specific legislation, performed
Belarus	Allowed	No	Allowed
Belgium	Allowed	No	No specific legislation, performed
Bulgaria	Allowed	Varies by region	Allowed
Czechia	Allowed	No	Allowed
Denmark	Allowed	Yes	No specific legislation, performed
Estonia	Allowed	No	Allowed
Finland	Allowed	Yes	No specific legislation, performed
France	Allowed	Yes	Allowed
Germany	Not allowed	–	Allowed
Greece	Allowed	No	Allowed
Hungary	Allowed	Yes	Not allowed
Iceland	Allowed	No	Allowed
Ireland	Allowed	–	No specific legislation, performed
Italy	Allowed	Varies by region	No specific legislation, performed
Japan	Not ruled	No	
Latvia	Allowed		No specific legislation, performed
Lithuania	Allowed	No	Not allowed
Malta	Allowed		Allowed
The Netherlands	Allowed	No	Allowed
Norway	Allowed	Yes	Allowed
Poland	Allowed	Varies by region	Allowed
Portugal	Allowed	Yes	No specific legislation, performed
Romania	Allowed	No	Allowed
Russia	Allowed	No	No specific legislation, performed
Serbia	Allowed	No	Not allowed
Slovakia	Allowed		Allowed
Slovenia	Allowed	Yes	Not allowed
South Korea	Not allowed		Allowed
Spain	ALLOWED	Varies by region	No specific legislation, performed
Sweden	Allowed	Varies by region	Allowed
United Kingdom	Allowed	Yes	Allowed

NOTE: I do not differentiate between contexts where only altruistic oocyte donation is allowed and countries where commercial oocyte donation is permitted. (See the Supporting Information for detailed sources.)

**TABLE 2:** Perceived upper age limit for motherhood and social importance of parenthood in European countries

	Adjusted average upper age limit for motherhood (years)			Parenthood is a social duty (% who agree)	
	2006–2007	2018–2019		2008–2010	2017–2020
Armenia				51.0	27.1
Poland	39.5	39.5		24.6	32.7
Romania				37.5	43.1
Serbia				30.4	28.2
Slovakia	38.8	38.2		28.0	41.9
Slovenia	40.4	41.7		24.0	23.8
Finland	40.8	42.2		7.7	9.5
Greece				37.1	
Iceland		42.4		7.1	7.7
Austria	42.4	43.1		21.5	25.2
Belgium	40.5	41.5		8.5	
Czechia		39.2		40.4	50.3
France	42.1	41.9		12.5	16.2
Germany	40.5	42.1		23.9	18.7
Denmark	39.9	41.1		14.1	12.7
Latvia		41.6		28.5	
Lithuania		41.7		24.2	40.9
The Netherlands	40.2	40.8		2.6	3.6
Portugal	41.6	39.0		40.0	37.0
Spain	41.6	39.4		24.7	24.0
Belarus				52.6	45.8
Bulgaria	37.8	41.0		63.8	77.3
Hungary	38.3	41.5		29.7	36.9
Russia	38.7			34.4	36.5
Estonia	40.6	42.6		26.4	24.4
Malta				42.4	
All countries	40.2	41.1		28.4	30.2

NOTE: Countries are sorted out based on their group in the constructed typology. SOURCE: European Social Survey (rounds 3 and 9) and European Values Study (waves 4 and 5).

**TABLE 3:** Age restrictions to IVF reimbursement eligibility and late fertility rates at the time of adoption of these policies

	≤42 (restrictive)	43+ or none (permissive)
≤0.8 Children per 100 women (low/moderate)	Alignment (Armenia, Poland, Romania, Serbia, Slovakia, Slovenia)	Anticipation (Belarus, Bulgaria, Hungary, Russia)
>0.8 Children per woman (high)	Lagging behind (Denmark, Japan, Latvia, Lithuania, Portugal, Spain)	Alignment (Finland, Iceland, Greece)
From low to high	Lagging behind (Austria, Belgium, Czechia, France, Germany)	Adaptation (Estonia, Malta, South Korea)

## Data Availability

Data on MAR legislation underlying this paper is available in the online Supporting Information. Access to the European Values Study (https://europeanvaluesstudy.eu/) and the European Social Survey (https://www.europeansocialsurvey.org/) is free of charge.

## Notes

1 Rather, the regulation of OD may have been governed by moral and ethical concerns regarding the status of eggs (Gunning 1998).

2 Reported age cutoffs are inclusive, that is, up to the specified age + 364 days. In general, age rules are the same for IVF, intra-cytoplasmic injections (ICSI) and intrauterine insemination (IUI), but they can differ in some countries. This is detailed in the online Supporting Information.

3 The number of IVF cycles which is publicly reimbursed can also be higher for IUI cycles (cf. Online Table A1).

4 Here, East Asia encompasses Japan, South Korea and Taiwan. CEE includes Armenia, Belarus, Bulgaria, Czechia, Estonia, Hungary, Poland, Russia, Romania, Serbia, Slovakia and Slovenia. Northern Europe corresponds to Denmark, Finland, Iceland, Norway and Sweden; Southern Europe to Greece, Italy, Malta, Portugal and Spain. Western European countries included in Figure 2 are Austria, Belgium, France, Germany, Ireland, the United Kingdom, the Netherlands and Switzerland.

5 For instance, in Austria in 2006–2007, the crude upper age limit for motherhood was 43.64 years old, acknowledged by 97.1 percent of respondents in this country. Therefore, the adjusted value is calculated as 43.64*0.971 = 42.37.

6 Overall, countries with strict age-based restrictions for reimbursement eligibility also limit the number of funded IVF cycles to a relatively strict number (up to four) and the other way around (permissive age-based rules associated with the reimbursement of five IVF cycles or more) (Online Table A1).

7 In Poland, a national reimbursement scheme partially covering treatments up to age 40 was only implemented between 2013 and 2016, before being left to regional authorities.

8 It has been argued that permissive regulations may be explained by the promotion of reproductive autonomy (Büchler and Parizer 2017), but explanations can also vary across countries of this group. In Greece, the increase in the direct age cutoff from 50 to 54 years old in 2022 was politically promoted as such and to adapt to changes in family formation behaviours (webteam 2022). A relatively large share of people in Greece also perceives parenthood as a social duty (37 percent in 2008–2010), while proportions are way lower in Iceland and Finland (below 10 percent). Pronatalist reasons may also motivate the permissiveness of regulations to allow people to access medical help to conceive. Commercial motivations have also been reported for Greece as many foreigners come to this country to benefit from fertility care at a cheaper cost than in other European contexts (Paraskou and George 2017). For the Nordic European countries of this group, the case of Finland also suggests regulatory permissiveness by default because of difficulties in reaching a legal consensus in drawing a law (Nordic Committee on Bioethics 2006).

9 For instance, 70 percent of medical costs are covered in Austria. France and Belgium fully cover IVF treatments. Public coverage was cut to 50 percent in 2004 in Germany (Figure 5). In these countries, as well as in Czechia, three to four IVF cycles are covered.

10 Latvia and Lithuania fully cover two IVF cycles. In Japan, the number of reimbursed IVF cycles varies with the women’s age (six for women below 40 years and four for women between 40 and 42 years old). Denmark only applied a cut in its coverage from 2010 to 2012 (Figure 6), otherwise fully reimburses three IVF cycles (including OD). In Portugal, the implementation of an age restriction for reimbursement was concomitant with the shift from partial to full coverage of three IVF cycles (Figure 6), which also includes OD (Table 1). Spain also covers three IVF cycles, but the extent of public reimbursement varies by provinces.

11 In that perspective, the coverage of infertility treatments shifted from partial to complete in Hungary in 2020 (dotted lines in Figure 7). It is partial in Belarus and full in Bulgaria and Russia.

12 Armenia is not displayed here because of missing information on ASFR45+ for several years.

13 The number of IVF cycles covered by reimbursement schemes also differs across countries of this group but without any apparent link with fertility trends. For instance, Slovenia fully covers up to six IVF cycles, while three cycles among childless women and two cycles for those who already had one child through IVF are covered in Serbia. The legislation of POC and OD also does not seem to explain differences in very late birth rates. OD is allowed and not publicly covered by national health plans in all countries of this group. POC is either allowed or not regulated.

14 France recently adopted a limit of 45 years old in 2021, replacing a soft access limit referred to as “childbearing ages.” IVF use has been limited up to age 45/47 in Belgium since 2007 and up to 49 in Czechia since 2011. Austria and Germany have no direct age limit to access IVF.

15 OD was allowed in the 2000s in Czechia and Belgium and is not allowed in Germany (Table 1). In Austria, it has been permitted since 2015 with an age limit of 45, an age-based restriction for egg donation that coexists with the absence of any age limit in the case of homologous fertilization (Calhaz-Jorge et al. 2020). Because success rates with gamete donation from a younger woman are higher than with a woman’s own eggs at late ages, this rule may be motivated by normative aspects rather than medical ones (Piek, Pennings, and Provoost 2023).

16 Because men’s fertility schedules are usually later than women’s, I plot ASFR45+ (Online Figure A1).
