# Supplementary material for: Alignment, Anticipation, Adaptation, or Lagging Behind? Age-Based Regulations in Assisted Reproduction and Late Fertility
Source: Popul Dev Rev. Author manuscript; Available in PMC 2024 Nov 15. (PMC7616811; doi:10.1111/padr.12658)
Supplement: Supplementary material [file EMS199896-supplement-Supplementary_material.pdf]

# Alignment, anticipation, adaptation or lagging behind? Age-based regulations in assisted reproduction and late fertility

## Supplementary material: Age-based regulations for assisted reproduction data

Table S1. The regulation of homologous IVF, planned oocyte cryopreservation and oocyte donation: timing, criteria and sources by country

| Country | IVF access (homologous) | | Public funding of IVF (homologous) | | | | | Planned oocyte cryopreservation (POC) and oocyte donation (OD) | | | | Sources |
|---|---|---|---|---|---|---|---|---|---|---|---|---|
| | Female age limit[a)] | Male age limit | Funding[b)] | Female age limit[c)] | Male age limit | Number of funded IVF cycles | Parities funded[d)] | OD allowed[e)] | Age limit for OD[f)] | OD publicly funded[g)] | POC allowed [h)] | |
| Austria | none (1992) | none (1992) | 70% (1999) | 39 (1999) | 49 (1999) | 4 (1999) | | yes (2015) | 45 (2015) | no | no | [1], [2] [3], [4] |
| Armenia | none (2002) | none (2002) | partial (2015) | 40 (2015) → 35/37 (2023) | none | 1/2 IVF (2023) | Childless / 1 child | yes | | no | not ruled, perform ed | [5] |
| Belarus | 50 (2012) | none | partial (between 2018 and 2021) | none | none | | | yes (2012) | | no | yes | [6], [7], [8] |

| | | | | | | | | | | | | |
|---|---|---|---|---|---|---|---|---|---|---|---|---|
| Belgium | 45/47 (2007) | none | complete (2003) | 42 (2003) | none | 6 (2003) | all | yes (2007) | | no | not ruled, perform ed | [1], [4], [8], [9], [10] |
| Bulgaria | 43 → none (2011) | none | complete (2009) | 43 | none | varies | | yes (2004) | | varies | yes | [3], [11], [12], [13] |
| Czechia | none (2006) → 49 (2011) | none | yes (1997) | 39 (1997) | none | 3-4 (2012) | | yes (2006) | | no | yes | [3], [11], [13], [14], [15], [16] |
| Denmark | 45 (1997) | none | complete | 40 (2006) | none | 3 | no own children (with IVF) | yes (1992) | | yes | yes | [11], [13], [17], [18], [19], [20] |
| Estonia | 50 (1997) | none | 50% (2004-17) → complete (2018) | 35 (2004) → 40 (2008) | none | 3 → unlimited (2008) | | yes | | no | yes | [11], [21], [22], [23] |
| Finland | none (2006) | none (2006) | partial | none | none | varies | | yes | | yes | not ruled, perform ed | [1], [6], [11], [17], [19], [24], [25] |
| France | ‘normal reproductive age’ (1994) → 45 (2021) | 59 (2021) | complete (1994) | 43 (1994) | 59 (2021) | 4 (1994) | | yes | | yes | yes (2021) | [1], [11], [20], [26] |

| | | | | | | | | | | | | |
|---|---|---|---|---|---|---|---|---|---|---|---|---|
| Germany | none (1990) | none | 100% → 50% (2004) | 39 (2004) | 49 (2004) | 4 → 3 (2004) | | no | | - | not ruled, performed | [1], [27] [4], [20] |
| Greece | 50 (2005) → 54 (2022) | none | partial | 50 | none | up to security funds | | yes | | no | yes (2022) | [1], [13], [28], [29], [30] |
| Hungary | 49 | none | partial (2014) → complete (2020) | 45 (2014) | none | 5 (2017) | no | yes | | yes | no | [11], [31], [32] |
| Iceland | ‘normal childbearing age’ (1996) | none (1996) | partial | none | none | | childless or 1 | yes | | no | yes | [19], [33], [34] [35] |
| Japan | not ruled | not ruled | yes (2004) | 42 (2016) | none | 6 (< 40) / 4 (40–42) (2016) | | not ruled | | no | | [6], [36], [37] |
| Latvia | none (2002) | none (2002) | complete (2012) | 37 (2012) | none | 2 | | yes | | no | yes | [6], [13], [25], [38], [39] |
| Lithuania | none (1999) → 45 (2017) | none | partial (2017) → complete (2021) | 42 (2017) | none | 2 | | yes (2016) | | no | no | [6], [40], [41] |
| Malta | none (2012) | none | yes (2013) | 42 → 45 (2022) | none | has been increased | childless couples | yes | | | yes (2018) | [42], [43] |

| | | | | | | | | | | | | |
|---|---|---|---|---|---|---|---|---|---|---|---|---|
| Poland | none (2015) | none | yes (2013 to 2016) > no | 40 (2013) → varies | none | | | yes | | varies | | [6], [44] |
| Portugal | none (2006) | none (2006) | partial (2009) → complete (2016) | 40 (2016) | | 3 | no ART child | yes | | | not ruled, performed | [1], [11], [13], [45], [46] |
| Romania | 48 | none | partial (2011) | 40 (2011) | none | | childless | yes | 50 | | yes | [11], [47] |
| Russia | none (1993) | none | complete (2013) | none | none | | | yes | | | yes | [11], [48], [49] |
| Serbia | none | none | complete (2006) | 42 (2006) | none | 3 (childless) / 2 (already one IVF child) | childless / one IVF child | yes | | no | yes | [50] |
| Slovakia | 35 (1983) → none | none | complete (2004) | 39 (2004) | none | 3 (2004) | | yes | | | yes | [51], [52] |
| Slovenia | ‘reproductive age’ (2000) | none | complete (2000) | 42 (2000) | none | 6 | | yes | | yes | no | [13], [25], [53] |
| South Korea | | | partial (2006) → 70% (2017) | 40 (2006) →44 (2017) → none (2019) | | 3 (2009) → 4 (2010-12) → 6 (2017) | | no | | | yes | [54], [55] |

| | | | | | | | | | | | | |
|---|---|---|---|---|---|---|---|---|---|---|---|---|
| | | | | | | → 7+5 (2019) | | | | | | |
| Spain | none (2006) | none | partial (1995) | 39 (2006) | 54 (2006) | 3 (2006) | 1 | yes | 50 | yes | yes | [1], [20], [56] |

## Notes by regulated areas:

a) <u>Female age limits for access to IVF:</u>

Women can begin IVF treatments up to the reported ages included [11].

Belgium: Egg retrieval is permitted up to age 45 and embryo transfer can be performed up to age 47 [11].
Bulgaria: There is an age limit of 51 years old according to [24], but none in other sources such as [13].
Iceland: The 1996 law mentions that a '*normal childbearing age*' refers to a couple. In 2008, Article 3 allowed access to IVF under the condition that '*the woman is of normal childbearing age and has the physical capacity and adequate health to cope with the stress associated with treatment, pregnancy and the birth of a child*' [57].
Malta: The 2012 law states that MAR entitlement is for '*any prospective parent* […] *provided that these procedures may only be resorted to where there is a reasonable chance of success and the procedures do not entail any known undue risk to the health of the woman or the child, beyond those already well-known as inherently associated with the procedure itself*' [43].
Portugal: There is no mention of an age limit in the law ([58], [1]) but 50 is often considered a 'reasonable age' and corresponds to the medical practice in private clinics [59]. This it is sometimes reported as the age cut-off to access IVF in some sources ([11], [13]).
Russia: There is no age limit according to [11], but a soft one referring to women in their '*childbearing age*' according to [49].
Slovakia: An age limit of 35 has been replaced by new legislation [51]. In 2013, it was reported that there is no legal age limit to access treatments [52]. In 2018, the age limit to access is said to be 50 [11].

b) <u>Publicly funded</u>:

This column indicates whether IVF treatments without any egg donation are partially or completely funded in the public sector. Note that public coverage can differ regarding medications. The rule can differ for intra-uterine insemination (IUI) or intra-cytoplasmic sperm injection (ICSI).

Armenia: According to [11], there was no public funding at all in 2018. Other sources report complete coverage for the first IVF attempt and 50% funding for the second one [5].
Austria: Does not cover IUI [6].
Belarus: Reimbursement schemes were adopted between 2018 and 2021 ([6], [8]). The existence of public funding in 2018 is reported in [11].
Bulgaria: Does not cover IUI [6]. The existence of some reimbursement schemes since 2017 is reported in [13].
Czechia: Does not cover ICSI [6]. The adoption of reimbursement schemes dates from 1982 according to [13] but 1997 according to [16].
Denmark: Complete coverage of IVF is reported in 1994 [19]. There was a cut between 2010 and 2011 in the full coverage (to 50%) [60]. Inconsistent reported information regarding whether the coverage is complete or partial in IFFS reports may be due to what is included in the reimbursement ([6], [8], [25], [30], [61]). Patients must pay for medication costs only according to [11]. Here, I rely on information from [13].
Finland: 80% covered in 1994 for IVF according to [19]. Partial according to IFFS 2004 and following reports ([6], [8], [25], [30], [61]).
Greece: Partial coverage in some IFFS reports ([25], [61]), complete in others ([62], [63]). There is no coverage according to the 2019 report [8]. According to IFFS 2022 [6], the coverage is partial as only fertility diagnoses and medications are covered. IVF, ICSI and IUI are not covered. The largest insurance company is governmental (National Organization for HealthCare Services Provision – EOPYY) and reimburses four IVF cycles until age 50 [64].
Iceland: Does not cover IUI [6]. The coverage depends on the number of IVF attempt ([19] for 1994 and [65] for 2004).
Japan: The reimbursement is partial and at the regional level ([6], [25]). It does not cover IUI [6] before 2020 [66].
Lithuania: Does not cover IUI [6].
Poland: Since 2016, only fertility diagnosis and medications are covered and this depends on the region [6].
Portugal: '*Medication and tests are only partially supported. The techniques are fully supported in public hospitals but are totally paid by couples in the private*' [63].
Romania: Does not cover IUI [6].
Russia: Does not cover IUI [6].
Slovakia: Cross-border reproductive care is covered, at least between 2012 and 2015 [30].
South Korea: Since 2006 (revised in 2009), partial coverage for low-income couples [55]. According to IFFS 2007, there is no coverage [62], but a partial one in the 2010 report [63]. There has been a national plan since 2017.
Spain: Partial coverage in some sources ([1], [6], [61], [63]) but complete in others ([8], [67]).

c) Female age limits for funding:

Women can be reimbursed if they begin IVF treatments up to the reported ages included [11].

Armenia: Women can be reimbursed up to age 38 if they have been registered in a fertility clinic at 35 or before. IUI is reimbursed up to age 42. For single women, as well as married couples who have lost a child during military service, the age limit is 42 [5].
Belgium: There were political debates about reducing the age limit to access funding from 42 to 41 years old [68]. One decree was passed in 2013 but was withdrawn a month later [69].
Denmark: Funding reported since 1994 [19].
Estonia: 40 years old since 1997 according to [13].
Finland: There is no mention of age restrictions in the 2006 law. According to [11], there are age limits of 40–45 for women and of 60 for men in practice.
Germany: Funding is restricted to IVF, married couples and without sperm donation [36].
Greece: None in 2018 according to [11].
Japan: Since 2016, up to six IVF cycles are funded for women under age 40, and up to four cycles for women aged 40–42 [36].
Malta: 48 (as for direct age limits) according to [11] but not to [42].
Slovakia: There is an age limit of 39 in 2013 according to [52] and 2022 [51], but none in 2009 according to [1].
Spain: Age limits are 'soft' [1] as regions have room to implement their own rules.

d) Parities publicly funded:

In some countries, eligibility for public reimbursement is conditional on the number of children couples/individuals already have [11]. When nothing is mentioned, there is no such criterion in the corresponding country.

Armenia: Treatment use is reimbursed for couples who have lost a child during military service, for childless couples who have been trying for more than two years and for married couples with already one child [5].
Denmark: IVF is not reimbursed for partners who already have had a child together. This does not apply if one of the partners already had children from a previous relationship. There is also no such limitation for the coverage of IUI [11].

e) OD:

This column refers to the situation in 2018 and relies on inforamation retrieved from [11]. I do not distinguish between altruistic and commercialised egg donation. In some countries such as Denmark, oocyte donation is only altruistic but egg donors can receive a financial compensation since 2006 [70]. In some countries, there are also age limits to be a donor (ex: 30 years old in Austria).

### f) <u>Age limit for OD:</u>

In the absence of any specification, the age limit to access OD is the same as homologous IVF.

### g) <u>Funding of OD:</u>

Data are from [6] and date back from 2021.

Spain: Not covered when women with medical issues are older than 36 years old [11]

### h) <u>POC:</u>

This column refers to the situation in 2018 and relies on information retrieved from [11], updated with data for 2021 [6]. In some countries, the legal availability of POC was confirming common practice (like in Greece) [11]. POC may be allowed or practised but not funded, like in Germany [4]. Conversely, it is publicly covered in France since 2021, but with age limits to freeze one's eggs (up to 37 years old). Countries can also limit the time people have to store their frozen eggs [71].

## References


[1] K. Berg Brigham, B. Cadier, and K. Chevreul, 'The diversity of regulation and public financing of IVF in Europe and its impact on utilization', *Human Reproduction*, vol. 28, no. 3, pp. 666–675, Mar. 2013, doi: 10.1093/humrep/des418.

[2] *IVF-Fonds-Gesetz*. 1999. [Online]. Available: https://www.ris.bka.gv.at/Dokumente/BgblPdf/1999_180_1/1999_180_1.pdf

[3] F. P. Busardò, M. Gulino, S. Napoletano, S. Zaami, and P. Frati, 'The evolution of legislation in the field of Medically Assisted Reproduction and embryo stem cell research in European union members', *Biomed Res Int*, vol. 2014, p. 307160, 2014, doi: 10.1155/2014/307160.

[4] N. Rimon-Zarfaty, J. Kostenzer, L.-K. Sismuth, and A. de Bont, 'Between "Medical" and "Social" Egg Freezing', *J Bioeth Inq*, vol. 18, no. 4, pp. 683–699, 2021, doi: 10.1007/s11673-021-10133-z.

[5] *Government of the Republic of Armenia Decision (ԻՐՏԵԿ - Իրավական տեղեկատվական կենտրոն)*. 2015. Accessed: Jul. 18, 2023. [Online]. Available: https://www.irtek.am/views/act.aspx?aid=80690

[6] IFFS, ‘International Federation of Fertility Societies’ Surveillance (IFFS) 2022: Global Trends in Reproductive Policy and Practice, 9th Edition’, *Global Reproductive Health*, vol. 7, no. 3, pp. e58–e58, 2022, doi: 10.1097/GRH.0000000000000058.
[7] *Law of the Republic of Belarus on assisted reproductive technologies (Закон Республики Беларусь О вспомогательных репродуктивных технологиях от 7 января 2012 г. № 341-З Содержание)*. 2012. Accessed: Jul. 26, 2023. [Online]. Available: https://etalonline.by/document/?regnum=H11200341#load_text_none_1_
[8] IFFS, ‘International Federation of Fertility Societies’ Surveillance (IFFS) 2019: Global Trends in Reproductive Policy and Practice, 8th Edition’, *Global Reproductive Health*, vol. 4, no. 1, pp. e29–e29, Mar. 2019, doi: 10.1097/GRH.0000000000000029.
[9] Service public federal sante publique, ‘Arrêté Royal du 04/06/2003 arrete royal modifiant l’arrete royal du 25 avril 2002 relatif a la fixation et a la liquidation du budget des moyens financiers des hopitaux’, etaamb.openjustice.be. Accessed: Sep. 29, 2023. [Online]. Available: https://etaamb.openjustice.be/fr/arrete-royal-du-04-juin-2003_n2003022627.html
[10] B. Lejeune, ‘Anonymat et compensation financière du don de gamètes et d’embryons en Belgique’, *Androl.*, vol. 18, no. 2, Art. no. 2, Jun. 2008, doi: 10.1007/BF03040396.
[11] C. Calhaz-Jorge *et al.*, ‘Survey on ART and IUI: legislation, regulation, funding and registries in European countries’, *Human Reproduction Open*, vol. 2020, no. 1, p. hoz044, Jan. 2020, doi: 10.1093/hropen/hoz044.
[12] R. Krastev and V. Mitev, ‘Correspondence Between Legislation and Public Opinion in Bulgaria about Access to Assisted Reproductive Technologies (ART)’, *Slovenian Journal of Public Health*, vol. 52, no. 4, pp. 285–291, Dec. 2013, doi: 10.2478/sjph-2013-0029.
[13] ‘International Reproduction Policy Database’. Accessed: Jul. 19, 2023. [Online]. Available: https://irpd.wzb.eu/data/
[14] J. Kocourková, B. Burcin, A. Stastna, and L. Šídlo, ‘The Impact of Assisted Reproduction on Fertility Trends in the Czech Republic’, presented at the International Population Conference, 2021.
[15] J. Kocourková, H. Konečná, B. Burcin, and T. Kučera, ‘How old is too old? A contribution to the discussion on age limits for assisted reproduction technique access’, *Reproductive BioMedicine Online*, vol. 30, no. 5, pp. 482–492, 2015, doi: 10.1016/j.rbmo.2015.01.017.
[16] J. Kocourková, A. Šťastná, and B. Burcin, ‘The influence of the increasing use of assisted reproduction technologies on the recent growth in fertility in Czechia’, *Sci Rep*, vol. 13, no. 1, Art. no. 1, Jul. 2023, doi: 10.1038/s41598-023-37071-7.
[17] T. Petersen, S. Soini, L. H. Guðmundsdóttir, J. Stoll, B. A. Faber, and for the Nordic Committee on Bioethics, ‘Legislation on biotechnology in the Nordic countries. An overview 2019’, presented at the NordForsk, Oslo, 2019. Accessed: Jul. 26, 2023. [Online]. Available: http://www.diva-portal.org/smash/get/diva2:1354854/FULLTEXT01.pdf
[18] Indenrigs- og Sundhedsministeriet, *Lov om kunstig befrugtning i forbindelse med lægelig behandling, diagnostik og forskning m.v.*, vol. LOV nr 460 af 10/06/1997. 1997. Accessed: Jul. 26, 2023. [Online]. Available: https://www.retsinformation.dk/eli/lta/1997/460

[19] M. Granberg, M. Wikland, and L. Hamberger, ‘Financing of IVF/ET in the Nordic countries’, *Acta Obstetricia et Gynecologica Scandinavica*, vol. 77, no. 1, pp. 63–67, 1998, doi: 10.1034/j.1600-0412.1998.770114.x.
[20] J. Gunning, ‘Oocyte donation: the legislative framework in Western Europe’, *Human Reproduction*, 1998, doi: 10.1093/HUMREP/13.SUPPL_2.98.
[21] A. Ehrenberg and E. Otter, ‘The regulation of ART in Estonia’, University of Tartu, 2018. Accessed: Jan. 25, 2023. [Online]. Available: http://ivfbaltic.eu/wp-content/uploads/2018/09/Aivar-Ehrenberg-The-regulation-of-assisted-reproduction_Estonia.pdf
[22] K. Allvee, M. Rahu, K. Haldre, H. Karro, and K. Rahu, ‘Quality of IVF status registration in the Estonian Medical Birth Registry: a national record linkage study’, *Reprod Health*, vol. 15, no. 1, p. 133, Dec. 2018, doi: 10.1186/s12978-018-0575-7.
[23] V. A. Hong Nguyen, ‘Descriptive study of the treatment cost of assisted reproductive technology in Estonia’, Master’s Thesis, Tallinn University of Technology, Tallinn, 2019.
[24] A. Büchler and K. Parizer, ‘Maternal Age in the Regulation of Reproductive Medicine – A Comparative Study’, *International Journal of Law, Policy and the Family*, vol. 31, no. 3, pp. 269–290, 2017.
[25] S. J. Ory, ‘IFFS Surveillance 2013’, 2007.
[26] Légifrance, *Décret n°2021-1243 du 28 septembre 2021 fixant les conditions d’organisation et de prise en charge des parcours d’assistance médicale à la procréation*. 2021. Accessed: Sep. 15, 2023. [Online]. Available: https://www.legifrance.gouv.fr/jorf/id/JORFTEXT000044111531
[27] O. Rauprich, E. Berns, and J. Vollmann, ‘Who should pay for assisted reproductive techniques? Answers from patients, professionals and the general public in Germany’, *Human Reproduction*, vol. 25, no. 5, pp. 1225–1233, May 2010, doi: 10.1093/humrep/deq056.
[28] E. Karakatsani, ‘Greece social briefing: Greece’s new law for Medical Assisted Reproduction’, China-CEE Institute, Budapest, Weekly Briefing 53 (3), 2022. [Online]. Available: https://china-cee.eu/2022/08/01/greece-social-briefing-greeces-new-law-for-medical-assisted-reproduction/#:~:text=Among%20key%20points%20introduced%20by,of%20the%20duration%20of%20the
[29] G. Leon, A. Papetta, and C. Spiliopoulou, ‘Overview of the Greek legislation regarding assisted reproduction and comparison with the EU legal framework’, *Reproductive BioMedicine Online*, vol. 23, no. 7, pp. 820–823, Dec. 2011, doi: 10.1016/j.rbmo.2011.07.024.
[30] ‘IFFS Surveillance 2016’, *Global Reproductive Health*, vol. 1, no. e1, pp. 1–143, Sep. 2016, doi: 10.1097/GRH.0000000000000001.
[31] D. Szekulesz, ‘“All planned babies must be born”: Women’s experience of infertility and assisted reproductive technologies in Hungary’, *Intersections. EEJSP*, vol. 8, no. 3, pp. 30–47, 2022.
[32] I. Szalma and T. Bitó, ‘Knowledge and attitudes about assisted reproductive technology: Findings from a Hungarian online survey’, *Reproductive BioMedicine and Society Online*, vol. 13, pp. 75–84, 2021, doi: 10.1016/j.rbms.2021.06.005.

[33] Nordic Committee on Bioethics, ‘Assisted Reproduction in the Nordic Countries. A comparative study of policies and regulation’, Nordic Council of Ministers, Copenhagen, 2006. Accessed: Dec. 17, 2021. [Online]. Available: http://public.ebookcentral.proquest.com/choice/publicfullrecord.aspx?p=3382713

[34] Ministry of Welfare, *Act on Artificial Fertilisation and use of Human Gametes and Embryos for Stem-Cell Research*. 2009. [Online]. Available: https://www.government.is/publications/legislation/lex/2009/08/26/Act-on-Artificial-Fertilisation-and-use-of-Human-Gametes-and-Embryos-for-Stem-Cell-Research-No.-55-1996/

[35] ‘Artificial Fertilization and Embryo Protection Act (Kunstliku viljastamise ja embrüokaitse seadus - KVEKS)’. Accessed: Jun. 21, 2023. [Online]. Available: https://www.riigiteataja.ee/akt/1048155?leiaKehtiv

[36] O. McDermott, L. Ronan, and M. Butler, ‘A comparison of assisted human reproduction (AHR) regulation in Ireland with other developed countries’, *Reprod Health*, vol. 19, no. 1, p. 62, Dec. 2022, doi: 10.1186/s12978-022-01359-0.

[37] Y. Hibino and S. Allan, ‘Absence of laws regarding sperm and oocyte donation in Japan and the impacts on donors, parents, and the people born as a result’, *Reproductive Medicine and Biology*, vol. 19, no. 3, pp. 295–298, 2020, doi: 10.1002/rmb2.12329.

[38] *Seksuālās un reproduktīvās veselības likums*. 2002. Accessed: Jul. 13, 2023. [Online]. Available: https://likumi.lv/doc.php?id=58982

[39] A. Grunskis, ‘The Legal Framework of Assisted Reproduction in Latvia’, Tartu, 2018. [Online]. Available: http://ivfbaltic.eu/wp-content/uploads/2018/09/Andris-Grunskis-The-regulation-of-assisted-reproduction_Latvia.pdf

[40] V. Mikelėnas and R. Mikelėnaitė, ‘Is the Battle Over? The New Lithuanian Law on Assisted Reproduction’, *Russ. law j.*, vol. 6, no. 1, pp. 119–132, Feb. 2018, doi: 10.17589/2309-8678-2018-6-1-119-132.

[41] A. Maslauskaite, ‘Lithuanian Families. Living diversity in times of outdated policies’, in *The Changing Faces of Families: Diverse Family Forms in Various Policy Contexts*, 1st ed., M. A. Adler and K. Lenz, Eds., London: Routledge, 2023, pp. 101–121. doi: 10.4324/9781003193500.

[42] F. Vella, ‘Estimating Models with Sample Selection Bias: A Survey’, *The Journal of Human Resources*, vol. 33, no. 1, p. 127, 1998, doi: 10.2307/146317.

[43] ‘LEĠIŻLAZZJONI MALTA’. Accessed: Nov. 21, 2023. [Online]. Available: https://legislation.mt/eli/act/2012/21/eng/pdf

[44] A. Janicka, R. Z. Spaczynski, K. Koziol, M. Radwan, and R. Kurzawa, ‘Assisted reproductive medicine in Poland, 2013–2016: Polish Society of Reproductive Medicine and Embryology (PTMRiE) and Fertility and Sterility Special Interest Group of the Polish Society of Gynaecologists and Obstetricians (SPiN PTGiP) report’, *Ginekol Pol*, vol. 92, no. 1, pp. 7–15, Jan. 2021, doi: 10.5603/GP.a2020.0142.

[45] D. da República, *Portaria n.º 154/2009*. Accessed: Nov. 23, 2023. [Online]. Available: https://diariodarepublica.pt/dr/detalhe/portaria/154-2009-601502

[46] *CIRCULAR NORMATIVA CONJUNTA ACSS/DGS*. 2016. [Online]. Available: https://www.acss.min-saude.pt/wp-content/uploads/2016/11/Normativa-4-2022-Acesso-a-Tratamentos-de-Procria%C3%A7%C3%A3o-Medic_DO_.pdf
[47] E. Brodeal, 'The Legal Status of Assisted Human Reproduction in Romania. A Brief Discussion on Surrogacy.', *Romanian Journal of Comparative Law*, vol. 1, pp. 56–74, 2016.
[48] T. K. Rostovskaya and O. V. Kuchmaeva, 'Assisted Reproductive Technologies through the Eyes of Russians', *Her. Russ. Acad. Sci.*, vol. 91, no. 5, pp. 578–586, Sep. 2021, doi: 10.1134/S1019331621050063.
[49] K. Svitnev, 'Legal regulation of assisted reproduction treatment in Russia', *Reproductive BioMedicine Online*, vol. 20, no. 7, pp. 892–894, Jun. 2010, doi: 10.1016/j.rbmo.2010.03.023.
[50] M. Rasevic and K. Sedlecky, 'Infertility and assisted reproductive technologies in Serbia', *STANOVNISHTVO*, vol. 60, no. 1, pp. 19–36, 2022, doi: 10.2298/STNV220418002R.
[51] V. Čunderlík Čerbová, 'A critical analysis of the legal regulation of assisted reproductive techniques and biomedical research in the Slovak Republic in the context of natural law', *Comparative Law Review*, vol. 28, 2022, doi: http://dx.doi.org/10.12775/CLR.2022.004.
[52] S. Karajičić, 'Policy on Assisted Reproduction in Slovakia', Health Policy Institute, Bratislava, Slovakia, 2013.
[53] *Zakon o zdravljenju neplodnosti in postopkih oploditve z biomedicinsko pomočjo (ZZNPOB)*. Accessed: Nov. 24, 2023. [Online]. Available: http://www.pisrs.si/Pis.web/pregledPredpisa?id=ZAKO2518
[54] I. Yun, W. Cha, C.-M. Nam, J. Y. Nam, and E.-C. Park, 'The impact of assisted reproductive technology treatment coverage on marriage, pregnancy, and childbirth in women of childbearing age: an interrupted time-series analysis', *BMC Public Health*, vol. 23, no. 1, p. 1379, Jul. 2023, doi: 10.1186/s12889-023-16286-3.
[55] J. Shin, S. G. Lee, E.-C. Park, and J. Y. Nam, 'Socioeconomic Status and Successful Delivery after an Infertility Diagnosis: a Nationwide Health Insurance Cohort Study in Korea Conducted from 2005 to 2013', *J Korean Med Sci*, vol. 35, no. 39, p. e341, 2020, doi: 10.3346/jkms.2020.35.e341.
[56] *Real Decreto 1030/2006, de 15 de septiembre, por el que se establece la cartera de servicios comunes del Sistema Nacional de Salud y el procedimiento para su actualización*. 2006. [Online]. Available: https://www.seg-social.es/wps/wcm/connect/wss/5604592f-be3c-4aeb-9adc-467b9eeb416f/4.RD_1030.2006.pdf?MOD=AJPERES
[57] '55/1996: Lög um tæknifrjóvgun og notkun kynfrumna og fósturvísa manna til stofnfrumurannsókna', Alþingi. Accessed: Nov. 21, 2023. [Online]. Available: https://www.althingi.is/lagas/nuna/1996055.html
[58] *Lei n.º 32/2006, de 26 de Julho*. Accessed: Nov. 23, 2023. [Online]. Available: https://diariodarepublica.pt/dr/detalhe/lei/32-2006-539239

[59] C. Neves, ‘Centros públicos com dois anos de atraso nos tratamentos de fertilidade’. Accessed: Nov. 23, 2023. [Online]. Available: https://www.dn.pt/sociedade/centros-publicos-com-dois-anos-de-atraso-nos-tratamentos-de-fertilidade-13333723.html

[60] J. Rothmar Herrmann, ‘Taming technology. Assisted reproduction in Denmark’, in *The Regulation of Assisted Reproductive Technologies in Europe. Variation, Convergence and Trends*, E. Griessler, L. Slepičková, H. Weyers, and N. Zeegers, Eds., in Routledge Studies in the Sociology of Health and Illness. , Routledge, Taylor & Francis Group, 2022, pp. 45–65.

[61] ‘IFFS Surveillance 2004’, *Fertility and Sterility*, vol. 81. Supplement 4, no. 5, 2004.

[62] ‘IFFS Surveillance 2007’, *Fertility and Sterility*, vol. 87, no. Sup 1, 2007.

[63] ‘IFFS Surveillance 2010’, *Fertility and Sterility*, 2010, doi: doi:10.1016/j.fertnstert.2010.08.011.

[64] E. Alexopoulou, P. Galanis, and K. Souliotis, ‘Assisted reproduction in Greece: Sociodemographic aspects and health behavior analysis’, *Popul. Med.*, vol. 4, no. June, pp. 1–9, Jun. 2022, doi: 10.18332/popmed/150331.

[65] H. S. Olafsdottir, W. Matts, and M. Anders, ‘Access to artificial reproduction technology in the Nordic countries in 2004’, *Acta Obstetricia et Gynecologica Scandinavica*, vol. 88, no. 3, pp. 301–307, 2009, doi: 10.1080/00016340802705949.

[66] Ministry of Health, Labour and Welfare, *Health insurance coverage of infertility treatments*. 2022. Accessed: Sep. 14, 2023. [Online]. Available: https://www.mhlw.go.jp/stf/seisakunitsuite/bunya/kodomo/kodomo_kosodate/boshi-hoken/funin-01.html

[67] ‘Policy of reimbursement - IVF-Worldwide’. Accessed: Nov. 04, 2022. [Online]. Available: https://ivf-worldwide.com/education/introduction/ivf-costs-worldwide/policy-of-reimbursement.html

[68] ‘L’âge limite du remboursement de la fécondation in vitro abaissé de 43 à 41 ans’, RTBF. Accessed: Jul. 26, 2023. [Online]. Available: https://www.rtbf.be/article/l-age-limite-du-remboursement-de-la-fecondation-in-vitro-abaisse-de-43-a-41-ans-7804194

[69] ‘Avis n°57 du 16 décembre 2013 relatif aux aspects éthiques de la congélation des ovules en prévision d’une infertilité liée à l’âge’.

[70] M. Storgaard, S. Malchau, A. Loft, E. Larsen, and A. Pinborg, ‘Oocyte donation is associated with an increased risk of complications in the pregnant woman and the fetus’, *Ugeskr Laeger*, vol. 179, no. 11, p. V11160817, Mar. 2017.

[71] V. M. L. Pedersen, ‘Freeze the Biological Clock: Discrimination, Disrespect, and Fertility Preservation via Social Freezing’, *Journal of Applied Philosophy*, vol. 39, no. 3, pp. 456–470, 2022, doi: 10.1111/japp.12572.
